# Engineering a minimal G protein to facilitate crystallisation of G protein-coupled receptors in their active conformation

**DOI:** 10.1093/protein/gzw049

**Published:** 2016-11-28

**Authors:** Byron Carpenter, Christopher G. Tate

**Affiliations:** MRC Laboratory of Molecular Biology, Cambridge Biomedical Campus , Francis Crick Avenue, CambridgeCB2 0QH, UK

**Keywords:** complex, G protein, G protein-coupled receptor, GPCR, G_s_, mini G protein, mini-G_s_

## Abstract

G protein-coupled receptors (GPCRs) modulate cytoplasmic signalling in response to extracellular stimuli, and are important therapeutic targets in a wide range of diseases. Structure determination of GPCRs in all activation states is important to elucidate the precise mechanism of signal transduction and to facilitate optimal drug design. However, due to their inherent instability, crystallisation of GPCRs in complex with cytoplasmic signalling proteins, such as heterotrimeric G proteins and β-arrestins, has proved challenging. Here, we describe the design of a minimal G protein, mini-G_s_, which is composed solely of the GTPase domain from the adenylate cyclase stimulating G protein G_s_. Mini-G_s_ is a small, soluble protein, which efficiently couples GPCRs in the absence of Gβγ subunits. We engineered mini-G_s_, using rational design mutagenesis, to form a stable complex with detergent-solubilised β_1_-adrenergic receptor (β_1_AR). Mini G proteins induce similar pharmacological and structural changes in GPCRs as heterotrimeric G proteins, but eliminate many of the problems associated with crystallisation of these complexes, specifically their large size, conformational dynamics and instability in detergent. They are therefore novel tools, which will facilitate the biochemical and structural characterisation of GPCRs in their active conformation.

## Introduction

G protein-coupled receptors (GPCRs) modulate cytoplasmic signalling, through heterotrimeric G proteins and β-arrestins, in response to extracellular stimuli, such as hormones and neurotransmitters (Rosenbaum *et al*., 2009). The central role of GPCRs in regulating cellular responses makes them an important therapeutic target (Lagerstrom and Schioth, 2008). GPCRs adopt different conformational states in response to binding different classes of ligand and coupling to cytoplasmic signalling proteins. Therefore, structure determination of GPCRs in all activation states is important to decipher the molecular mechanisms of signal transduction, and to facilitate optimal drug design.

Heterotrimeric G proteins are composed of α, β and γ subunits. Gα consists of a GTPase domain (GαGTPase), which is analogous to members of the small GTPase superfamily of proteins, and an α-helical domain (GαAH), which is unique to heterotrimeric G proteins (Sprang, 1997). In the inactive, GDP-bound state, Gα binds Gβγ, forming a heterotrimer with low basal nucleotide exchange activity (Higashijima *et al*., 1987). The trimer is anchored to the cell membrane, through lipid modifications of both Gα and Gγ (Spiegel *et al*., 1991). GPCRs catalyse rapid nucleotide exchange on heterotrimeric G proteins, but only weakly activate the isolated α subunit (Herrmann *et al*., 2006; Phillips *et al*., 1992).

Agonist binding to a GPCR promotes its transition to a structural state that can efficiently interact with heterotrimeric G proteins (Lebon *et al*., 2011b; Rasmussen *et al*., 2011b; Rosenbaum *et al*., 2011; Xu *et al*., 2011). The agonist-bound receptor engages the C-terminal region of Gα (Hamm *et al*., 1988), initiating a rotation and displacement of the α5 helix (Oldham *et al*., 2006). This ultimately destabilises the nucleotide-binding pocket and the GαGTPase–GαAH domain interface, allowing GDP to dissociate (Alexander *et al*., 2014; Dror *et al*., 2015; Flock *et al*., 2015; Kaya *et al*., 2014; Sun *et al*., 2015; Van Eps *et al*., 2011). The resulting nucleotide-free ternary complex displays large, mutually induced structural changes in both the receptor and G protein (Rasmussen, *et al*., 2011b), and is often characterised by increased agonist binding affinity of the receptor (Delean *et al*., 1980). This complex can be trapped in the absence of guanine nucleotides (Bornancin *et al*., 1989), but is extremely short-lived *in vivo* due to rapid binding of GTP to Gα (Vuong *et al*., 1984). GTP binding triggers dissociation of the G protein from the receptor (Kuhn, 1981) and separation of Gα from Gβγ (Fung *et al*., 1981).

GPCR–G protein complexes are difficult targets for structural studies due to their large size, conformational dynamics, and instability in detergent. To date, only a single structure of a GPCR–G protein complex has been reported, namely the β_2_-adrenergic receptor (β_2_AR) bound to the adenylate cyclase stimulating G protein G_s_ (Rasmussen *et al*., 2011b). This structure provided the first atomic resolution insight into the organisation of the ternary complex, but further structures are required to fully decipher the molecular mechanisms of signal transduction and the specificity for G protein coupling. Given the difficulties in crystallising GPCR–G protein complexes, novel tools are needed to facilitate their high-throughput crystallisation.

Here, we report the design of a minimal G protein, termed mini-G_s_, which is composed solely of the GαGTPase domain from G_s_. Mini-G_s_ closely mimics the pharmacological and structural changes induced in GPCRs by heterotrimeric G_s_. It is therefore a novel tool, which will facilitate the characterisation of GPCRs in their active conformation, and has allowed the structure determination of the adenosine A_2A_ receptor in the fully active state (Carpenter *et al*., 2016).

## Materials and Methods

### Cloning

Details of G protein, β_1_AR and mini-G_s_ constructs used in this work are provided in Supplementary Tables SI–SIII, respectively. All G proteins used in this study were mutated to remove sites of lipid modification. G protein cDNAs were cloned into the transfer vector pBacPAK8 (Clontech), and baculoviruses were prepared using the flashBAC ULTRA system (Oxford Expression Technologies). Synthetic genes (Integrated DNA Technologies) for Nb80 (Rasmussen *et al*., 2011a) and Nb35 (Westfield *et al*., 2011) were cloned into pET26b (Novagen) for periplasmic expression in *Escherichia* *coli*. Mini-G_s_ constructs, which were derived from the long isoform of the human Gα_s_ gene, were cloned into the pET15b vector (Novagen) for expression in *E. coli*.

### Expression and purification of β_1_AR

β_1_AR constructs were expressed in insect cells using the baculovirus expression system, and purified as described previously (Warne *et al*., 2003; Warne *et al*., 2011; Warne *et al*., 2009).

### Baculovirus expression of G proteins

*Trichoplusia ni* cells (Expression Systems) were grown in ESF921 serum-free media (Expression Systems) in 5 L optimum growth flasks (Thompson Instrument Company). Immediately before infection, heat-inactivated foetal bovine serum (Sigma) was added to a final concentration of 5%. Cells were infected with third passage virus at a final concentration of 3%. In the case of co-infection with multiple viruses (for heterotrimeric G_s_ or Gβγ) each virus was added to a final concentration of 3%. The final volume of culture was 3 L per flask and the final cell density was 3 × 10^6^ cells/ml. Cells were harvested 48 h post-infection by centrifugation at 5000 *g* for 5 mins, flash-frozen in liquid nitrogen and stored at −80°C.

### Protein purification

Details of G protein, nanobody and mini-G_s_ purifications are provided in the Supplementary material.

### Saturation binding assay

Insect cell membranes containing β_1_AR were resuspended in assay buffer (20 mM HEPES pH 7.5, 100 mM NaCl). The sample was aliquoted and [^3^H]-dihydroalprenolol was added (to give final concentrations in the range of 0.25 nM to 256 nM), alprenolol was added to the negative control (1 mM final concentration). Samples were incubated at 20°C for 2 h, before filtering through 96-well glass fibre filter plates (Merck Millipore) and washing with ice-cold assay buffer. Radioactivity was quantified by scintillation counting and apparent *K*_*d*_ values were determined using GraphPad Prism version 5.0 (GraphPad Software, San Diego, CA).

### Competition binding assay

Insect cell membranes containing β_1_AR were resuspended in assay buffer (25 mM HEPES pH 7.5, 100 mM NaCl, 1 mM MgCl_2_, 1 mM ascorbate). The sample was aliquoted and binding partner (25 μM final concentration), isoprenaline (final concentrations in the range of 1 pM–100 mM) and apyrase (0.1 U/ml final concentration) were added. Alprenolol was added to the negative control (100 μM final concentration). Samples were incubated at 20°C for 1.5 h, before adding [^3^H]-dihydroalprenolol (5 or 20 nM final concentrations for β_1_AR^ΔNC^ or β_1_AR-84, respectively). Samples were incubated at 20°C for 1.5 h, before filtering through 96-well glass fibre filter plates and washing with ice-cold assay buffer. Radioactivity was quantified by scintillation counting and *K*_*i*_ values were determined using GraphPad Prism version 5.0.

Competition binding assays using detergent-solubilised β_1_AR-84 were performed using a similar protocol, except: all steps were performed at 4°C; membranes were solubilised with dodecyl maltoside (DDM; 0.1% final concentration) for 30 min, prior to addition of binding partner and ligands; separation of bound from free ligand (by gel filtration) was performed exactly as described in the thermostability assay protocol (below).

### Thermostability measurement of **β**_1_AR_∆NC_–mini-Gs complexes

Thermostability assays were performed using a modified version of previously described methods (Lebon *et al*., 2011a; Serrano-Vega *et al*., 2008). Insect cell membranes containing β_1_AR^∆NC^ were resuspended in assay buffer (25 mM HEPES pH 7.5, 400 mM NaCl, 1 mM MgCl_2_, 1 mM ascorbate, 0.1% BSA, 0.004% bacitracin). The sample was aliquoted and binding partner (25 μM final concentration), ^3^H-norepinephrine (200 nM final concentration) and apyrase (0.1 U/ml final concentration) were added. Norepinephrine was added to the negative control (200 μM final concentration). Samples were incubated at 4°C for 1 h, before solubilisation with detergent for 1 h on ice. The detergents dodecyl maltoside (DDM), decyl maltoside (DM), nonyl glucoside (NG) or octyl glucoside (OG) were used at final concentrations of 0.1, 0.13, 0.3 or 0.8%, respectively. Samples were heated to different temperatures (between 4 and 50°C) for exactly 30 min, followed by quenching on ice for 30 min. Samples were separated by gel filtration through Toyopearl HW-40F resin packed in a 96-well filter plate (Merck Millipore). Radioactivity was quantified by scintillation counting and apparent melting temperature (*T*_*m*_) values were determined using GraphPad Prism version 5.0.

### Thermostability measurement of GDP-bound mini-G_s_ mutants by differential scanning fluorimetry

Differential scanning fluorimetry (DSF) was performed essentially as described previously (Niesen *et al*., 2007). Mini-G_s_ mutants (30 μg) were diluted with assay buffer (10 mM HEPES pH 7.5, 100 mM NaCl, 1 mM MgCl_2_, 1 mM GDP, 2 mM DTT). SYPRO-orange was added to give a final concentration of ×2. Thermostability measurements were performed using a Rotor-Gene Q (Qiagen). Samples were equilibrated for 90 s at 25°C before ramping from 25 to 99°C at 4 s/°C. The apparent melting temperature (*T*_*m*_), corresponding to the inflection point of the curve, was derived from analysis using the Rotor-Gene Q software.

### Gel filtration analysis of mini G protein complexes

The mini-G_s_–βγ complex was prepared using mini-G_s_399, a construct in which the N-terminal residues 6–25 were replaced and the L272D mutation was reversed (Supplementary Table SIII). Purified mini-G_s_399 was mixed with non-lipidated Gβ_1_γ_2_ dimer in an equimolar ratio and incubated on ice for 4 h. The sample was loaded onto a Superdex-200 10/300 gel filtration column (GE healthcare), equilibrated with gel filtration buffer (10 mM HEPES pH 7.5, 100 mM NaCl, 1 mM MgCl_2_, 1 μM GDP, 0.1 mM TCEP).

The β_1_AR–mini-G_s_ complex was prepared using β_1_AR^∆NC^ purified in lauryl maltose neopentyl glycol (LMNG) detergent. Purified β_1_AR^∆NC^ was mixed with a 1.2-fold molar excess of mini-G_s_393 and incubated on ice for 4 h. The sample was loaded onto a Superdex-200 10/300 gel filtration column, equilibrated with gel filtration buffer (10 mM HEPES pH 7.5, 100 mM NaCl, 1 mM MgCl_2_, 1 μM ascorbic acid, 1 μM isoprenaline, 0.002% LMNG). Peak fractions were analysed by SDS-PAGE on a 4–20% Tris-glycine gel (Thermo Fisher).

The gel filtration column was calibrated using molecular weight standards (Sigma), and the apparent molecular weight of samples was calculated using the calibration curve shown in Supplementary Fig. S9.

### Statistical analysis

Unpaired, two-tailed *t*-tests were used to compare two data sets, and *P* values are quoted in the text.

## Results

### Strategy to develop a minimal G protein

**Fig. 1:**
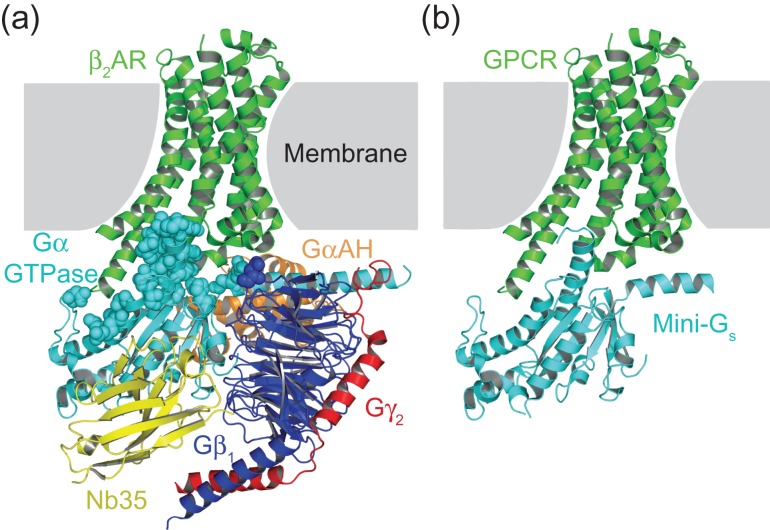
Design of a minimal G protein. (**a**) Crystal structure of the β_2_AR–G_s_ complex (PDB code 3SN6; Rasmussen *et al*., 2011b). The intracellular component of this complex, which is composed of Gα_s_, Gβ_1_, Gγ_2_ and Nb35, totals over 100 kDa in molecular weight. However, over 97% of direct contacts (3.9 Å cut-off) between β_2_AR and G_s_ are formed by the GαGTPase domain (cyan). Residues from G_s_ that form contacts with β_2_AR are shown as spheres. (**b**) Model of the proposed complex between a GPCR and mini-G_s_ (isolated GαGTPase domain). The intracellular component of this complex is a single protein with a molecular weight of approximately 27 kDa. Figures were prepared using PyMOL (The PyMOL Molecular Graphics System, Version 1.7.4 Schrödinger, LLC)

The aim of this work was to isolate the minimum component of G_s_ that could be used to crystallise native-like GPCR–G protein complexes. The molecular weight of G_s_ is 90 kDa, however, the β_2_AR–G_s_ complex (Rasmussen *et al*., 2011b) revealed that more than 97% of direct contacts between the G protein and receptor are mediated by the 27 kDa GαGTPase domain (Fig. 1a). We hypothesised that this domain would be sufficient to stabilise GPCRs in their fully active state, i.e. the conformation adopted by β_2_AR in the β_2_AR–G_s_ complex (Rasmussen *et al*., 2011b), and it was therefore used as the starting point to engineer a minimal G protein (mini-G_s_; Fig. 1b).

Three binding partners were used as controls during this work: Nb80, a nanobody that binds to β_2_AR and induces a comparable shift in agonist binding affinity to heterotrimeric G_s_ (Rasmussen *et al*., 2011a); soluble heterotrimeric G_s_ that was mutated to remove all potential lipidation sites, referred to herein as G_s_ (Supplementary Table SI); and Nb35, a nanobody that stabilises G_s_ in its GPCR-bound conformation (Westfield *et al*., 2011). G_s_ was either used alone (referred to as G_s_) or in the presence of Nb35 (referred to as G_s_–Nb35). As active state structures of β_2_AR bound to either Nb80 or G_s_–Nb35 have been determined (Rasmussen *et al*., 2011a, 2011b), the stability and pharmacological activity of an engineered mini-G_s_ should, at a minimum, reflect the analogous properties of these binding partners.

The β_1_-adrenergic receptor (β_1_AR) was used as a model GPCR in the development of mini-G_s_. Both β_1_AR and β_2_AR are able to bind G_s_ and Nb80 and exhibit a significant increase in agonist binding affinity. Either receptor could have been used for the development of mini-G_s_, but because our laboratory has developed a large number of thermostabilised β_1_AR variants (Miller and Tate, 2011; Serrano-Vega *et al*., 2008), we chose to work with β_1_AR. We did not use the adenosine A_2A_ receptor for the development of mini-G_s_, because there is only a small increase in agonist affinity upon binding a G protein (Carpenter *et al*., 2016).

To be most useful for the characterisation and structure determination of GPCRs in their active state, mini-G_s_ needed to fulfil a number of criteria. Mini-G_s_ should be stable enough in its basal conformation to allow high-yield expression and purification, promote the transition of β_1_AR to the high-affinity agonist-bound state, and form a stable complex with detergent-solubilised β_1_AR. The development of mini-G_s_ involved a number of key steps, some of which only became apparent as the work progressed: (i) development of a sensitive assay to detect G protein coupling to β_1_AR; (ii) isolation of the GαGTPase domain (mini-G_s_) and demonstration of binding to β_1_AR; (iii) thermostabilisation of the β_1_AR–mini-G_s_ complex in membranes; (iv) thermostabilisation of the β_1_AR–mini-G_s_ complex in detergent; and (v) validation of the final mini-G_s_ construct. Details of each of these steps are given under the corresponding subheadings later.

### Development of a sensitive assay to detect G protein coupling to **β**_1_AR

A sensitive competition binding assay was developed that could detect the interaction of different binding partners with β_1_AR, by measuring the affinity of agonist binding to the receptor. A heterologous competition format was used to determine the agonist binding affinity (*K*_*i*_) of β_1_AR by measuring binding of the antagonist ^3^H-dihydroalprenolol (^3^H-DHA; Supplementary Fig. S1) in the presence of increasing concentrations of the agonist isoprenaline. The concentration of binding proteins used in the assays was standardised to 25 μM, which was approximately 30-fold above the equilibrium dissociation constant (*K*_*D*_) for Nb80 binding to β_1_AR (Miller-Gallacher *et al*., 2014). Initially, we used a truncated form of turkey β_1_AR (Warne *et al*., 2003), which was designated β_1_AR^∆NC^ (Supplementary Table SII). This construct did not contain any thermostabilising mutations, and behaved identically to full-length receptor in cell-signalling assays (Baker *et al*., 2011), despite containing truncations of disordered regions in the N-terminus and C-terminus. The *K*_*i*_ for isoprenaline binding to β_1_AR^∆NC^ was 40 ± 0 nM in the absence of a binding partner, which shifted to 5.8 ± 0.8 nM, 17 ± 2 nM or 6.8 ± 0.6 nM in response to Nb80, G_s_ or G_s_–Nb35, respectively (Supplementary Fig. S2). The shift in isoprenaline *K*_*i*_ upon G protein binding to β_1_AR^∆NC^ was relatively small, which was unsuitable for detecting potentially small changes elicited upon binding of unstable G protein derivatives during the development of mini-G_s_. We therefore used a minimally thermostabilised β_1_AR construct (β_1_AR-84; Supplementary Table SII), which contained four mutations that increased the stability preferentially of the inactive state of the receptor (Miller-Gallacher *et al*., 2014; Warne *et al*., 2011), in addition to the truncations at the N-terminus and C-terminus. β_1_AR-84 had a lower affinity for isoprenaline (*K*_*i*_ of 2.6 ± 0.3 μM) in its uncoupled state than β_1_AR^∆NC^, but showed a larger shift in agonist binding affinity when coupled to either Nb80, G_s_ or G_s_–Nb35 (*K*_*i*_ of 28 ± 1 nM, 271 ± 54 nM or 16 ± 4 nM, respectively; Table I and Supplementary Fig. S2). The competition binding data fitted best to single-site binding parameters. Therefore, the partial shift in isoprenaline *K*_*i*_ observed for some binding partners most likely reflected incomplete stabilisation of the high-affinity agonist-bound state, rather than indicating partial coupling or mixed receptor populations. Although G_s_ was able to couple β_1_AR, Nb35 was required to stabilise the G_s_ complex, resulting in β_1_AR–G_s_–Nb35 complexes with similar affinity for isoprenaline compared to the β_1_AR–Nb80 complexes (Table I and Supplementary Fig. S2). The competition binding assay using β_1_AR-84 showed a 162-fold increase in isoprenaline affinity upon G_s_–Nb35 coupling to the receptor, which was far larger than the 6-fold shift in affinity induced by G_s_–Nb35 binding to β_1_AR^∆NC^. We therefore used β_1_AR-84 in all subsequent competition binding assays during the development of mini-G_s_.

**Table I.** β_1_AR-84 competition binding data

Binding partner	Mutation	CGN code^a^	β_1_AR-84 isoprenaline *K*_*i*_ (nM)		Effect on expression
			4°C	20°C	
None	n.a.^b^	n.a.	2100 ± 180 (*n* = 12)	2600 ± 270 (*n* = 15)	n.a.
Nb80	n.a.	n.a.	n.d.^c^	28 ± 1 (*n* = 2)	n.a.
G_s_	n.a.	n.a.	420 ± 80 (*n* = 2)	271 ± 54 (*n* = 2)	n.a.
G_s_–Nb35	n.a.	n.a.	n.d.	16 ± 4 (*n* = 3)	n.a.
Mini-G_s_77	Parental	n.a.	99 ± 12 (*n* = 4)	1900 ± 230 (*n* = 3)	n.a.
	H41I	41^G.S1.2^	32	390	=
	H41V	41^G.S1.2^	51	490	=
	A48L	48^G.s1h1.2^	43	170	=
	G49D	49^G.s1h1.3^	25	280	=
	E50N	50^G.s1h1.4^	37	720	=
	R201A	201^G.hfs2.2^	31	1480	−
	G226A	227^G.s3h2.2^	86	820	=
	227–230 sub^d^	227^G.s3h2.3^	23	530	=
	E230A	230^G.H2.3^	51	540	−
	A249D	249^G.S4.7^	10	35	+
	A249E	249^G.S4.7^	70	390	=
	S252D	252^G.s4h3.3^	14	94	+
	S252E	252^G.s4h3.3^	38	380	+
	255–264 del^e^	254^G.s4h3.5^	21	20	+
	L272D	272^G.H3.8^	7	310	=
	L272E	272^G.H3.8^	28	750	=

Competition binding data showing the isoprenaline *K*_*i*_ of β_1_AR-84 in the presence of different binding partners. Data are from a single experiment performed in duplicate unless otherwise stated; where two or more independent experiments were performed, data represent mean ± SEM, from the number (*n*) of independent experiments performed in duplicate. The effect of mutations on the expression level of mini-G_s_ was estimated from SDS-PAGE gels. Expression levels are described as being equal to (=), more than 2-fold lower than (−), or more than 2-fold higher than (+) the parental construct.

^a^Common Gα numbering (CGN) system (Flock *et al*., 2015).

^b^Not applicable.

^c^Not determined.

^d^Substitution of switch II residues 227–230 with two glycine residues.

^e^Deletion of switch III residues 255–264.

### Isolation of the G**α**_s_ GTPase domain and measuring binding to **β**_1_AR-84

The GαGTPase domain from Gα_s_ had previously been expressed as an isolated protein, in order to determine its role in guanine nucleotide binding and hydrolysis (Markby *et al*., 1993), but its ability to couple to GPCRs had never been investigated. We isolated the GTPase domain by replacing the sequence corresponding to GαAH with a short glycine linker (Supplementary Table SIII). This construct, mini-G_s_77, expressed poorly in *E. coli* and could not be purified to homogeneity, indicating that it was very unstable. Nevertheless, a small amount of partially pure protein could be prepared (Supplementary Fig. S3), and was tested for its ability to couple β_1_AR-84 (at 20°C) in either the presence or absence of Gβγ–Nb35. No significant shift in the isoprenaline *K*_*i*_ of β_1_AR-84 (2.6 ± 0.3 μM) was observed in the presence of mini-G_s_77 (1.9 ± 0.2 μM; *P* = 0.254), but mini-G_s_77–Gβγ–Nb35 induced a large shift in isoprenaline affinity to 3.6 ± 0.8 nM (*P* = 0.004; Table I and Supplementary Fig. S2). Thus partially purified mini-G_s_77 was functional, but the data also suggested that it was unable to couple β_1_AR-84 in the absence of Gβγ when assayed at 20°C. In contrast, when the assay was performed at 4°C mini-G_s_77 induced a significant shift in isoprenaline *K*_*i*_ from 2.1 ± 0.2 μM for uncoupled β_1_AR-84 (at 4°C) to 99 ± 12 nM (*P* < 0.001; Table I and Supplementary Fig. S2). This demonstrated that the isolated GαGTPase domain (mini-G_s_77) could couple to β_1_AR-84 in the absence of Gβγ, but suggested that the thermostability of the GαGTPase domain was a limiting factor in its ability to stabilise the high-affinity agonist-bound state of the receptor.

### Thermostabilisation of the **β**_1_AR–mini-G_s_ complex in membranes

**Fig. 2:**
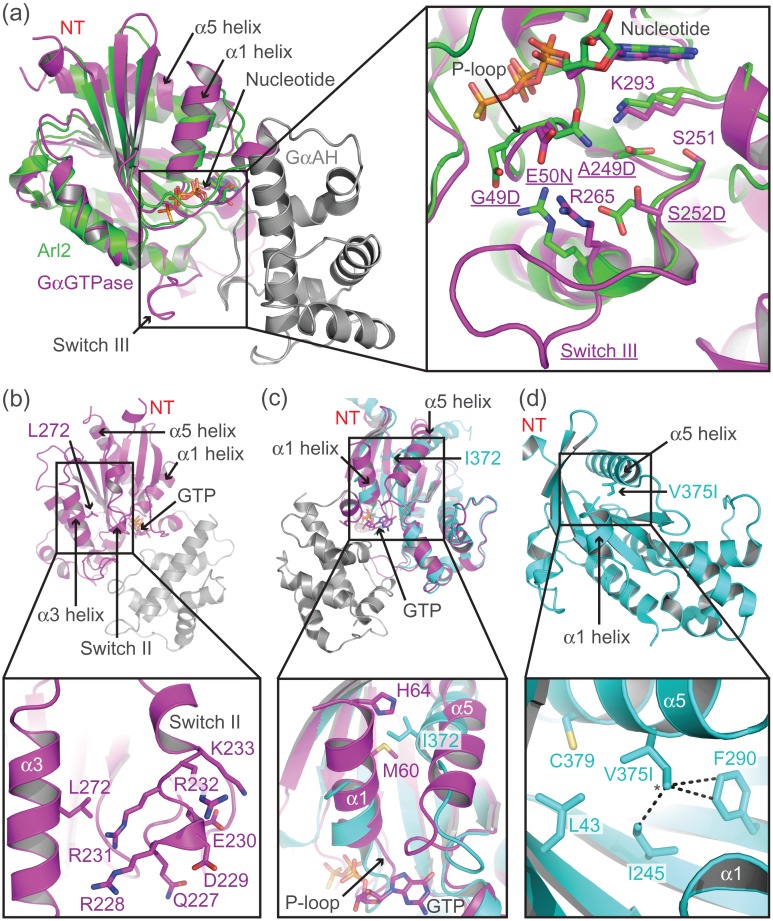
Rational design of mutations to stabilise mini-G_s_. (**a**) Structural alignment of Gα_s_ (PDB code 1AZT; Sunahara *et al*., 1997), coloured magenta and grey, and Arl2 (PDB code 1KSH; Hanzal-Bayer *et al*., 2002), coloured green. The Gα_s_ GTPase domain aligns to Arl2 with an RMSD of 1.9 Å, despite sharing sequence identity of only 25%, determined using the Dali server (Holm and Rosenstrom, 2010). See Supplementary Fig. S4 for a sequence alignment between Gα_s_ and Arl-2. The inset shows an expanded view of mini-G_s_ residues (shown as sticks and underlined name) that were mutated (G49D, E50N, A249D, and S252D) to match the corresponding residue in Arl2. Residues with which the mutations potentially interact are shown as sticks. (**b**) Mutation of Leu272, which is located within the α3 helix of Gα_s_ (PDB code 1AZT; Sunahara *et al*., 1997), to aspartic acid allows potential interactions with a cluster of charged and polar residues (227–233) in the N-terminal region of switch II. (**c**) Alignment of Gα_s_ in its GTP-bound conformation (PDB code 1AZT; Sunahara *et al*., 1997), coloured magenta, and GPCR-bound conformation (PDB code 3SN6; Rasmussen *et al*., 2011b), coloured cyan. In the GPCR-bound conformation Ile372 (α5 helix) sterically clashes with Met60 and His64 (α1 helix), preventing close packing of the α1 helix against the core of the GαGTPase domain. (**d**) The V375I mutation (modelled using PyMOL) was designed to increase hydrophobic contacts between the core of the GαGTPase domain and the α5 helix in its GPCR-bound conformation (PDB code 3SN6; Rasmussen *et al*., 2011b). Residues that interact with Val375 are shown as sticks, additional contacts (less than 4.2 Å), which are predicted to be formed by the δ-carbon (*) of the isoleucine mutation are displayed as dashed lines.

Rational design mutagenesis was employed to thermostabilise mini-G_s_ in complex with membrane-embedded β_1_AR. Mutations were designed based on structural alignments (Fig. 2a and Supplementary Fig. S4) between Gα_s_ (Sunahara *et al*., 1997) and Arl2 (Hanzal-Bayer *et al*., 2002); Arl2 is the small GTPase with the greatest structural similarity to Gα_s_. This initial mutagenic screen primarily targeted regions of Gα_s_ that were close to the GαAH domain interface or that were known to be conformationally dynamic. Mutants were screened using the competition binding assay at both 4 and 20°C. Due to the low, and variable expression level of the mutants, it was not easy to standardise the concentration of mini-G_s_ mutants used in the assays. Therefore, the total mini-G_s_ purified from 1 L of *E. coli* culture was used per competition curve (Table I). Approximately 100 mutants were tested during this initial screen. Mutations that shifted the isoprenaline *K*_*i*_ of β_1_AR-84 more than 2-fold compared to the parental mini-G_s_ construct (mini-G_s_77) at either temperature were classed as positive. A total of 16 positive mutations, covering 12 unique positions were identified (Table I).

Some of the single mini-G_s_ mutants produced a near maximal shift in the agonist binding affinity of β_1_AR-84 in the competition binding assay, therefore, mutation combinations could not be reliably tested using this assay, as further small increases in agonist affinity would be difficult to accurately measure. Instead, the mutation combinations were tested in complex with detergent-solubilised β_1_AR^∆NC^ using a thermostability (*T*_*m*_) assay (Table II). The agonist ^3^H-norepinephrine (^3^H-NE) was used in the *T*_*m*_ assay, however due to the high background signal associated with this ligand, a maximum concentration of 200 nM could be used. This was approximately equal to the *K*_*i*_ of uncoupled β_1_AR^∆NC^, but approximately 250-fold above the *K*_*i*_ of β_1_AR^∆NC^ complexed with Nb80 or G_s_–Nb35 (Supplementary Fig. S5). Therefore, apparent *T*_*m*_ values quoted for uncoupled β_1_AR^∆NC^ are under non-saturated agonist conditions, but β_1_AR^ΔNC^ complexes, which have higher agonist binding affinity, are under agonist-saturated conditions.

**Table II.** β1AR_∆NC_ thermostability data

Binding partner	Mutation	CGN code^a^	Apparent *T*_*m*_ of β_1_AR^∆NC^ in DDM, measured by ^3^H-NE binding (°C)	Stability of GDP-bound mini-G_s_ measured by DSF (°C)
None	n.a.^b^	n.a.	25.9 ± 0.0 (*n* = 3)	n.a.
Nb80	n.a.	n.a.	32.0 ± 0.0 (*n* = 3)	n.a.
G_s_–Nb35	n.a.	n.a.	35.8 ± 0.1 (*n* = 3)	n.a.
Gα_s_	n.a.	n.a.	n.d.^c^	50.1 ± 0.1 (*n* = 3)
Mini-G_s_162	A249D		25.1 (*n* = 1)	60.6 ± 0.1 (*n* = 3)
Mini-G_s_164	A249D, SIII^d^		28.6 (*n* = 1)	66.5 ± 0.0 (*n* = 3)
Mini-G_s_165	A249D, S252D, SIII		28.5 ± 0.2 (*n* = 2)	68.7 ± 0.0 (*n* = 3)
Mini-G_s_169	A249D, S252D, SIII, L272D		28.8 (*n* = 1)	67.1 ± 0.0 (*n* = 3)
Mini-G_s_183	G49D, E50N, A249D, S252D, SIII, L272D		28.7 ± 0.2 (*n* = 4)	72.5 ± 0.0 (*n* = 3)
Mini-G_s_199^e^	G49D, E50N, A249D, S252D, SIII, L272D		29.2 ± 0.2 (*n* = 17)	72.5 ± 0.0 (*n* = 3)
Mini-G_s_254	Mini-G_s_199 + M60A	60^G.H1.8^	31.5 ± 0.3 (*n* = 5)	70.3 ± 0.0 (*n* = 3)
Mini-G_s_350	Mini-G_s_199 + L63Y	63^G.H1.11^	30.9 ± 0.4 (*n* = 2)	70.7 ± 0.0 (*n* = 3)
Mini-G_s_340	Mini-G_s_199 + I372A	372^G.H5.4^	34.0 (*n* = 1)	66.6 ± 0.1 (*n* = 3)
Mini-G_s_303	Mini-G_s_199 + V375I	375^G.H5.7^	31.5 ± 0.6 (*n* = 3)	70.3 ± 0.0 (*n* = 3)
Mini-G_s_352	Mini-G_s_199 + L63Y, I372A		34.5 (*n* = 1)	64.7 ± 0.1 (*n* = 3)
Mini-G_s_345	Mini-G_s_199 + I372A, V375I		35.0 (*n* = 1)	65.4 ± 0.1 (*n* = 3)
Mini-G_s_393^f^	Final mini-G_s_ construct		34.1 ± 0.5 (*n* = 3)	65.3 ± 0.0 (*n* = 3)

Thermostability data for either detergent-solubilised β_1_AR^∆NC^ complexes or mini-G_s_ mutants in the GDP-bound state. Apparent *T*_*m*_ values represent the mean ± SEM from the number (*n*) of independent experiments performed in duplicate. Some apparent *T*_*m*_ values were determined from a single experiment, with an assumed error of ±0.5°C. Apparent *T*_*m*_ values for mini-G_s_ in the GDP-bound state were determined by differential scanning fluorimetry (Supplementary Fig. S6).

^a^Common Gα numbering (CGN) system (Flock *et al*., 2015).

^b^Not applicable.

^c^Not determined.

^d^Deletion of switch III residues 255–264 is referred to as SIII.

^e^Mini-G_s_199 contains the same mutations as mini-G_s_183, but has a redesigned linker region (Supplementary Table SIII), and was used as the parental construct for screening detergent-stabilising mutations.

^f^Mini-G_s_393 contains the same mutations as mini-G_s_345, but has an additional truncation of the N-terminus and redesigned linker region (Supplementary Table SIII), and was used as the starting construct for crystallisation trials.

A new parental construct (mini-G_s_161), was used to test combinations of the mutations; mini-G_s_161 contained a larger deletion encompassing GαAH with a slightly longer linker than in mini-G_s_77 (Supplementary Table SIII). The A249D mutant (mini-G_s_162) produced the largest shift in the agonist binding affinity of membrane-embedded β_1_AR-84 in the competition binding assay, however, in detergent the β_1_AR^∆NC^–mini-G_s_162 complex had an apparent *T*_*m*_ of 25.1°C (Table II), which was lower than that of uncoupled of β_1_AR^∆NC^ (25.9°C). Addition of the switch III deletion, which induced the second largest shift in the agonist binding affinity of membrane-embedded β_1_AR-84, to make a double mutant (mini-G_s_164), increased the apparent *T*_*m*_ of the complex to 28.6°C (Table II). However, this was still lower than that of either β_1_AR^∆NC^–Nb80 or β_1_AR^∆NC^–G_s_–Nb35, by 3.4 and 7.2°C, respectively (Table II). Addition of other mutations that were classed as positive in the competition binding assay failed to further increase the apparent *T*_*m*_ of the β_1_AR^∆NC^–mini-G_s_ complex (examples shown in Table II), indicating that the complex was particularly unstable in detergent. This was confirmed by the observation that mini-G_s_ mutants were unable to shift the agonist binding affinity of detergent-solubilised β_1_AR-84 to the same degree as that of membrane-embedded β_1_AR-84 in competition binding assays (results not shown). Despite the fact that they did not further stabilise the β_1_AR^∆NC^–mini-G_s_ complex in detergent, four additional mutations (G49D, E50N, S252D and L272D) were added to mini-G_s_164 to produce the construct mini-G_s_183 (Table II). These mutations were utilised because they all individually increased the isoprenaline *K*_*i*_ of membrane-embedded β_1_AR-84 (Table I). They also increased the stability of the basal GDP-bound state mini-G_s_183 by 6°C compared to mini-G_s_164 (Table II and Supplementary Fig. S6), as assessed by differential scanning fluorimetry.

Five of the six mutations that were combined in mini-G_s_183 were clustered around the nucleotide-binding pocket and phosphate-binding loop (P-loop; Fig. 2a). The A249D mutation was designed to interact with Lys293 and Ser251, in order to stabilise the base of the nucleotide-binding pocket. Deletion of switch III was intended to stabilise mini-G_s_, by replacing this flexible loop with the defined secondary structure elements (α-helix, 3_10_-helix and β-turn) found in Arl2 (Hanzal-Bayer *et al*., 2002). The S252D mutation was also designed to stabilise the region around switch III, through a potential interaction with Arg265. The G49D and E50N mutations, which are located in the P-loop, were designed to reduce flexibility and conformationally constrain this region, through potential interactions with Arg265 and Lys293, respectively. The sixth mutation (L272D) was designed to conformationally constrain switch II, through potential interactions with a cluster of charged and polar residues (227–233) within its N-terminal region (Fig. 2b).

### Thermostabilisation of the **β**_1_AR_∆NC_–mini-G_s_ complex in detergent

The majority of mutations from the first mutagenic screen did not stabilise the β_1_AR–mini-G_s_ complex in detergent. We hypothesised that this was because they did not specifically stabilise mini-G_s_ in it receptor-bound conformation, therefore, a second panel of approximately 150 mutants were designed with the intention of stabilising the receptor-bound conformation of mini-G_s_. This mutagenic screen was based on the structure of the β_2_AR–G_s_ complex (Rasmussen *et al*., 2011b), and focused on regions of Gα_s_ that undergo large conformational changes upon receptor binding. Many of the mutations tested during this second screen were destabilising to mini-G_s_ in its basal GDP-bound state, therefore they could not be tested individually (i.e. in the mini-G_s_161 parental construct). Instead, they were added to mini-G_s_199, which was identical to mini-G_s_183, except that it contained a modified linker region (Supplementary Table SIII). This construct was very stable in its basal GDP-bound state (72.5°C) and could thus negate the destabilising effects of the additional mutations. Mini-G_s_ mutants were screened in complex with detergent-solubilised β_1_AR^∆NC^ using the ^3^H-norepinephrine *T*_*m*_ assay. Four stabilising mutations were identified (Table II), the best of which (I372A) increased the apparent *T*_*m*_ of the complex from 29.2 to 34.0°C and it combined additively with V375I, to give an apparent *T*_*m*_ of 35.0°C. This was 3.0°C higher than the β_1_AR^∆NC^–Nb80 complex and only 0.8°C lower than the β_1_AR^∆NC^–G_s_–Nb35 complex. The other mutations did not combine additively with I372A and V375I and were rejected (results not shown).

Both of the positive detergent-stabilising mutations were located within the α1 or α5 helices. Alignment of Gα_s_ in its receptor-bound conformation (Rasmussen *et al*., 2011b) with the GTP-bound structure (Fig. 2c; Sunahara *et al*., 1997) identified an unfavourable steric clash across the α1–α5 helix interface, involving residues Met60, His64 (from the α1 helix) and Ile372 (from the α5 helix). This clash was predicted to prevent close packing of the C-terminal region of the α1 helix against the α5 helix and the core of the GαGTPase domain, thus exposing the core of the protein to the solvent. The I372A mutation was designed to eliminate this clash and facilitate better packing in this region. Similarly, the V375I mutation was designed to improve packing between the α5 helix and the core of the protein in its receptor-bound conformation (Fig. 2d).

### Validation of mini-G_s_

**Fig. 3:**
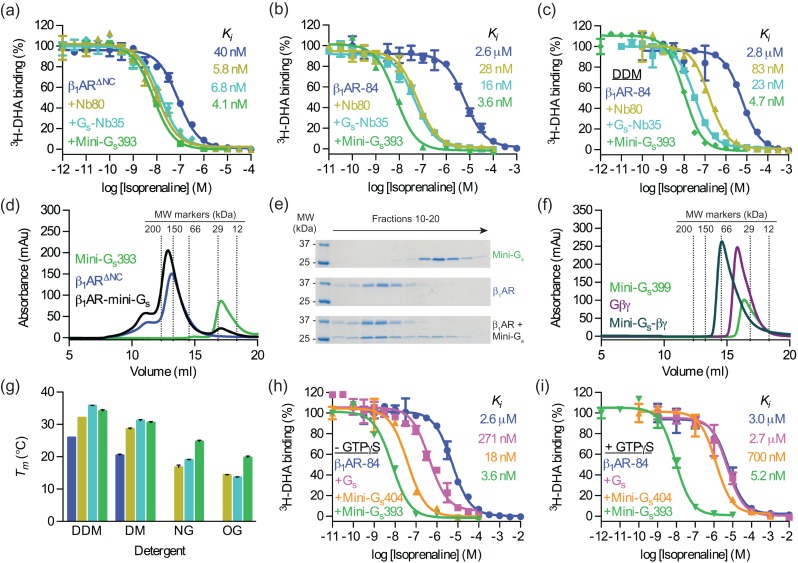
Validation of mini-G_s_ (**a–c**) A competition binding assay was used to measure the change in affinity (*K*_*i*_) of isoprenaline induced by Nb80, G_s_–Nb35, or mini-G_s_393 coupling to: (a) membrane-embedded β_1_AR^∆NC^, (**b**) membrane-embedded β_1_AR-84 and (**c**) DDM-solubilised β_1_AR-84. (**d**) Analytical gel filtration analysis of β_1_AR^∆NC^ binding to mini-G_s_393. The apparent molecular weight of mini-G_s_393 was 23 kDa (17.1 ml), which compares well with the theoretical value of 27 kDa. The apparent molecular weight of β_1_AR^∆NC^ was 139 kDa (13.2 ml), which is consistent with the 45 kDa receptor being associated with a large detergent micelle; the shoulder at 11 ml (> 300 kDa) probably represents aggregated receptor. A mixture of β_1_AR^∆NC^ and mini-G_s_393 (1.2-fold molar excess) resolved as a predominant peak with an apparent molecular weight of 160 kDa (12.9 ml). The 21 kDa increase in the apparent molecular weight of the β_1_AR^∆NC^–mini-G_s_393 complex compared to uncoupled β_1_AR^∆NC^ is consistent with mini-G_s_393 binding with 1:1 stoichiometry. (**e**) SDS-PAGE analysis of the gel filtration eluate confirmed the presence of both β_1_AR^∆NC^ and mini-G_s_393 in the peak fractions. (**f**) Analytical gel filtration analysis of Gβγ binding to mini-G_s_399. The apparent molecular weights of mini-G_s_399 (Supplementary Table SIII) and Gβγ were 32 kDa (16.4 ml) and 42 kDa (15.8 ml), respectively, which is in close agreement with the theoretical values of 29 kDa and 46 kDa, respectively. An equimolar mixture of mini-G_s_399 and Gβγ resolved as a single peak with an apparent molecular weight of 73 kDa (14.6 ml). The 31 kDa increase in the apparent molecular weight of the mini-G_s_399–Gβγ complex compared to Gβγ is consistent with mini-G_s_399 binding with 1:1 stoichiometry. (**g**) Thermostability of detergent-solubilised β_1_AR^∆NC^ alone or in complex with Nb80, G_s_–Nb35, or mini-G_s_393, in different detergents (Supplementary Fig. S10). Uncoupled β_1_AR^∆NC^ did not survive solubilisation in NG or OG. Colours correspond to those used in (a). (**h–i**) GTP-mediated dissociation of β_1_AR-84 complexes, measured by competition binding assay. The response in isoprenaline *K*_*i*_ induced by G_s_, mini-G_s_404 (Supplementary Table SIII), or mini-G_s_393 coupling to β_1_AR-84 was measured in the presence or absence of GTPγS (250 μM). (a–c,h,i) Data are representative of at least two independent experiments, each performed in duplicate, with error bars ± SEM. (g) Data represent mean ± SEM of at least two independent experiments, each performed in duplicate.

The detergent-stabilised construct (mini-G_s_345) was modified for crystallographic applications by changing the linker and shortening the N-terminus (Supplementary Table SIII). The final stabilised construct, mini-G_s_393 (Supplementary Fig. S7 and S8), was able to elicit an equal or greater shift in isoprenaline affinity compared to either Nb80 or G_s_–Nb35 (Fig. 3a–c) whether the experiments were performed using membrane-embedded β_1_AR^∆NC^ (*K*_*i*_ of 4.1 ± 1.1 nM compared to 5.8 ± 0.8 nM and 6.8 ± 0.6 nM, respectively), membrane-embedded β_1_AR-84 (*K*_*i*_ of 3.6 ± 0.0 nM compared to 28 ± 1 nM and 16 ± 4 nM, respectively) or detergent-solubilised β_1_AR-84 (*K*_*i*_ of 4.7 ± 0.4 nM compared to 83 ± 2 nM and 23 ± 7 nM, respectively). Crucially, for the mini-G_s_393 complex, high-affinity isoprenaline binding was maintained when β_1_AR-84 was solubilised in detergent, and was 17-fold higher than that of the Nb80 complex and 5-fold higher than that of the G_s_–Nb35 complex. Thus mini-G_s_393 is an ideal protein for the formation of stable GPCR complexes in detergent solution.

The biochemical properties of mini-G_s_393 also make it ideal for structural studies of GPCR complexes. Mini-G_s_393 was readily purified to homogeneity with a yield of 100 mg of purified protein per litre of *E. coli* culture, and it could be concentrated to over 100 mg/ml (Supplementary Fig. S9). Analytical gel filtration showed that mini-G_s_393 bound to purified β_1_AR^∆NC^ in LMNG (Fig. 3d and e), demonstrating that the complex could be purified in detergent. Recent innovations in electron cryo-microscopy (cryo-EM; Bai *et al*., 2015) suggest that large GPCR–G protein complexes could be amenable for structure determination by single particle imaging. It is therefore useful to note that mini-G_s_399, a construct in which the N-terminal residues 6–25 were replaced and the L272D mutation was reverted to wild type (Supplementary Table SIII), retained its ability to form a heterotrimer with Gβγ (Fig. 3f). The main advantage of the mini-G_s_399–Gβγ complex over heterotrimeric G_s_, is that it lacks the GαAH domain, which is highly dynamic in the GPCR-bound conformation (Westfield *et al*., 2011). Therefore GPCR complexes composed of mini-G_s_399–Gβγ are predicted to be more conformationally homogenous than those involving heterotrimeric G_s_, and thus better suited to cryo-EM applications.

Crystallisation of GPCRs often requires the use of short chain detergents, so the thermostability of the β_1_AR^∆NC^–mini-G_s_393 complex was tested in four different detergents using the ^3^H-norepinephrine *T*_*m*_ assay and compared to the analogous complexes with Nb80 and G_s_–Nb35 (Fig. 3g and Supplementary Fig. S10). Under all conditions tested uncoupled β_1_AR^∆NC^ was significantly less stable than when bound to either mini-G_s_393, Nb80 or G_s_–Nb35. Similarly, β_1_AR^∆NC^ was always more stable bound to mini-G_s_393 compared to Nb80. β_1_AR^∆NC^–mini-G_s_393 complexes were also considerably more stable than β_1_AR^∆NC^–G_s_–Nb35 complexes in short chain detergents, although the later was marginally more stable in DDM. The striking improvement in the thermostability by 5–8°C of the β_1_AR^∆NC^–mini-G_s_393 complex in NG and OG in comparison to other complexes suggests it has significant advantages for the structure determination of receptors in the active state.

The nucleotide-binding properties of the mutants were not extensively studied in this work, but one interesting observation was that the β_1_AR-84–mini-G_s_393 complex was resistant to dissociation by physiological concentrations of GTP (Fig. 3h and i). GTPγS fully reversed the shift in *K*_*i*_ induced by G_s_ binding to β_1_AR-84 from 271 ± 54 nM to 2.7 ± 0.1 μM. The shift in isoprenaline *K*_*i*_ induced by mini-G_s_404 (an identical construct to mini-G_s_393, except that the I372A and V375I mutations were reverted to wild type; Supplementary Table SIII) binding to β_1_AR-84 was almost fully reversed by GTPγS (from 18 ± 2 nM to 700 ± 60 nM). However, there was no significant difference in the isoprenaline *K*_*i*_ of the β_1_AR-84–mini-G_s_393 complex in either the presence or absence of GTPγS (3.6 ± 0.0 nM compared to 5.2 ± 0.7 nM; *P* = 0.137). This unresponsiveness to GTPγS was caused by the I372A mutation (see Discussion), because mini-G_s_391 (an identical construct to mini-G_s_393, except that only the V375I mutation was reverted to wild type; Supplementary Table SIII), behaved in a similar fashion to mini-G_s_393 (Supplementary Fig. S11). Unresponsiveness to GTP is a useful property that should allow the formation of stable GPCR–mini-G_s_ complexes *in vivo*, which may be a novel method to improve expression and purification of unstable GPCRs.

## Discussion

Several novel approaches have been developed to stabilise and crystallise GPCRs in their active conformation, including complexation with G protein-derived peptides (Scheerer *et al*., 2008), G protein-mimicking nanobodies (Huang *et al*., 2015; Kruse *et al*., 2013; Rasmussen *et al*., 2011a; Ring *et al*., 2013) and a nanobody-stabilised heterotrimeric G protein (Rasmussen *et al*., 2011b). All of these complexes appear to stabilize the receptor in its active state, which is characterised by an outward movement of helix 6 and conserved conformational changes of residues within the core of the receptor, particularly R^3.50^, Y^5.58^ and Y^7.53^ (Huang *et al*., 2015). The β_2_AR–G_s_ complex provided the first insight into the organisation of the native GPCR–G protein interface, which is something that other binding proteins cannot recreate, but frustratingly, complexes involving heterotrimeric G_s_ are also the most difficult to crystallise, due to their large size and dynamic nature. Therefore, we designed a minimal G protein that offers significant advantages to the crystallisation of native-like GPCR–G protein complexes, specifically mini-G_s_ is a small, soluble, highly expressed protein, which readily forms a detergent-stable complex with GPCRs.

Our lab has previously determined the structures of both β_1_AR and the adenosine A_2A_ receptor (A_2A_R) bound to agonists (Lebon *et al*., 2011b; Warne *et al*., 2011), and therefore crystallisation trials were conducted for both β_1_AR and A_2A_R in complex with mini-G_s_, employing a parallel approach of lipidic cubic phase (LCP) and vapour diffusion. Crystals were obtained quickly for wild type A_2A_R in complex with mini-G_s_ by vapour diffusion in the detergent octylthioglucoside (OTG), and we were able to solve the structure to 3.4 Å resolution (Carpenter *et al*., 2016). β_1_AR crystallisation trials are still at an early stage, and have not yet yielded crystals that diffract to sufficient resolution for structure determination. The molecular organisation of A_2A_R–mini-G_s_ is remarkably similar to that of the β_2_AR–G_s_ complex (Rasmussen *et al*., 2011b), with A_2A_R adopting a conformation that closely resembles G_s_-bound β_2_AR. In addition, mini-G_s_ bound to A_2A_R is very similar to the analogous region in G_s_ bound to β_2_AR (Rmsd 0.9 Å; Carpenter *et al*., 2016). Thus mini G proteins are useful surrogates for heterotrimeric G proteins to stabilise and determine structures of GPCRs in their active conformation. However, it must be appreciated that GPCRs have not evolved to be stable in the activated state bound to a G protein, but instead have evolved to be unstable so that signalling lasts for short, defined, periods of time before being terminated. Therefore, additional approaches, such as thermostabilisation of the GPCR (Tate, 2012) bound to mini-G_s_, may be required before the structures of many complexes can be determined. The high-level expression and stability of mini-G_s_ makes this a trivial undertaking compared to using the wild type heterotrimeric G protein. It is also important to note that further modifications of mini-G_s_, such as deletions and mutations, may be required to facilitate crystallisation of different GPCRs. The crystal structure of the A_2A_R–mini-G_s_ complex was solved using mini-G_s_414, which is identical to mini-G_s_393 except that it contains the additional mutation L63Y (Table II). Well diffracting crystals of the A_2A_R–mini-G_s_ complex were grown using either mini-G_s_393 or mini-G_s_414, however, the A_2A_R–mini-G_s_414 complex produced a different crystal form that diffracted to slightly better resolution, and was thus used for structure determination. There was no discernible difference between these two complexes in either the competition binding assay or thermostability assay (results not shown), suggesting that the main effect of the L63Y mutation was on crystallogenesis.

**Fig. 4:**
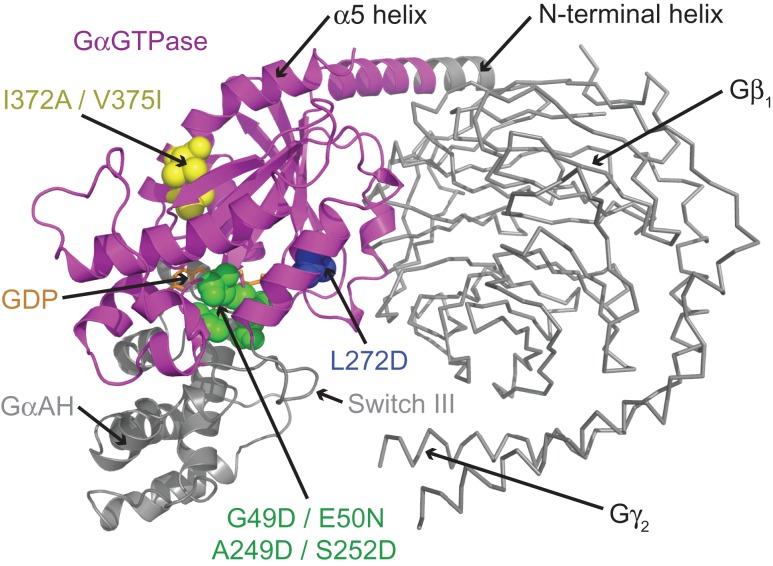
A model of heterotrimeric G_s_ highlighting the region that corresponds to mini-G_s_ (magenta). The model of heterotrimeric G_s_ was constructed by superposition of the crystal structures of Gα_s_ (PDB code 1AZT; Sunahara *et al*., 1997) and heterotrimeric Gα_t/i1_ (PDB code 1GOT; Lambright *et al*., 1996). Residues that were mutated in mini-G_s_ (shown as spheres) were clustered in three regions of the protein: the nucleotide-binding pocket (green), switch II (blue), and the α5 helix (yellow). Regions of Gα_s_ that were deleted in mini-G_s_ (GαAH, switch III and half of the N-terminal helix) are coloured grey. The Gβγ subunits, which are not required for mini-G_s_ coupling to GPCRs are shown as ribbons and coloured grey. GDP is shown as sticks and coloured orange.

The development of mini-G_s_ involved extensive screening to identify key deletions and point mutations that improved both the conformational homogeneity and thermostability of the β_1_AR–mini-G_s_ complex (Fig. 4). Deletion of the GαAH domain, which is the most dynamic region of G_s_ in its GPCR-bound conformation, significantly reduced the conformational heterogeneity in the β_1_AR–mini-G_s_ complex. This deletion also eliminated the requirement of Gβγ subunits for GPCR coupling, removing the need for exogenous components, such as Nb35, to stabilise the Gα–Gβγ interface. In the absence of Gβγ subunits the N-terminus of mini-G_s_ could be partially deleted, resulting in a more compact protein. The switch III deletion removed a dynamic region of mini-G_s_, replacing it with defined secondary structure components, and resulted in a significant improvement in the thermostability of the complex. A total of seven point mutations were required to fully stabilise mini-G_s_ in complex with β_1_AR (Fig. 4). Extensive mutagenic screens were employed that targeted most regions of mini-G_s_, however, all of the mutations utilised in the final construct were clustered around three regions of the protein (nucleotide-binding pocket, switch II, and α5 helix). The G49D, E50N, A249D and S252D mutations were designed to stabilise the nucleotide-binding pocket and P-loop (Fig. 2a), and all of these mutations improved the thermostability of both the basal GDP-bound state of mini-G_s_ and the β_1_AR–mini-G_s_ complex. The L272D mutation, which was located adjacent to switch II, was designed to conformationally constrain this flexible region (Fig. 2b), and resulted in improved thermostability of the β_1_AR–mini-G_s_ complex. The I372A and V375I mutations, which were located in the α5 helix, were designed to improve packing between the core of protein and the α1 or α5 helices, respectively (Fig. 2c and d). These two mutations specifically improved the thermostability of the β_1_AR–mini-G_s_ complex in detergent. None of these mutations were located within the receptor-binding site, ensuring that the native GPCR–G protein interface was maintained, and allowing mini-G_s_ to be co-crystallised with other G_s_-coupled receptors. During the course of this work the I372A mutation was also independently reported to stabilise a complex between rhodopsin and the adenylate cyclase inhibiting G protein G_i1_ (Sun *et al*., 2015).

The G protein engineering work has also provided insight into the mechanism of G protein activation. Binding of Gα_s_ to β_2_AR triggers displacement of the G protein α5 helix to a position that is predicted to sterically clash with the α1 helix. This unfavourable clash appears to prevent close packing of the C-terminal region of the α1 helix against the α5 helix and the core of the GTPase domain (Fig. 2c), and this region is indeed disordered in the β_2_AR–G_s_ complex (Rasmussen *et al*., 2011b). The α1 helix forms part of the nucleotide-binding pocket and directly connects to the P-loop (Fig. 2c), which is the main determinant of nucleotide binding affinity in G proteins (John *et al*., 1990). Destabilisation of the α1 helix has previously been suggested to be a key event in receptor-mediated nucleotide exchange (Flock *et al*., 2015; Kaya *et al*., 2014; Sun *et al*., 2015), however the mechanism of this destabilisation was unclear. Here, we identified a potential steric clash between Ile372 from the α5 helix and residues from the α1 helix (in particular Met60), which appears to play an important role in this nucleotide exchange. Mutation of Ile372 to alanine, which was predicted to eliminate the steric clash, was shown to inhibit GTP-mediated dissociation of the β_1_AR–mini-G_s_ complex. These data indicate that, in G_s_, Ile372 acts as a relay between the GPCR-binding site (α5 helix) and the key regions of the nucleotide-binding pocket (α1 helix and P-loop), allowing the receptor to allosterically destabilise the nucleotide-binding pocket and modulate nucleotide exchange. We suggest that the I372A mutation uncouples GPCR binding from occupancy of the nucleotide-binding pocket, a hypothesis that was supported by the presence of GDP in one of the two copies of mini-G_s_ in the asymmetric unit of the A_2A_R–mini-G_s_ structure (Carpenter *et al*., 2016).

Mini G proteins are novel tools that have many potential applications, including characterisation of receptor pharmacology in response to different classes of agonists (full, partial and weak), binding affinity and kinetic studies, thermostabilisation of GPCRs in their active conformation, drug discovery, and structure determination of native-like GPCR–G protein complexes. Furthermore, all of the mutations reported here are located within conserved regions of the Gα subunit. Therefore, the concept is potentially transferable to all classes of heterotrimeric G proteins, which would allow the production of a panel of mini G proteins capable of coupling any GPCR.

## Supplementary Material

Supplementary Data
