# Supplementary material for: Engineering a minimal G protein to facilitate crystallisation of G protein-coupled receptors in their active conformation
Source: Protein Eng Des Sel. 2016 Nov 28;29(12):583–94. doi: 10.1093/protein/gzw049 (PMC5181381; doi:10.1093/protein/gzw049)
Supplement: Supplementary Data [file supp_29_12_583__index.html]

Engineering a minimal G protein to facilitate crystallisation of G protein-coupled receptors in their active conformation — Engineering a minimal G protein to facilitate crystallisation of G protein-coupled receptors in their active conformation — Supplementary Data 

# Engineering a minimal G protein to facilitate crystallisation of G protein-coupled receptors in their active conformation

## Supplementary Data

Supplementary Data

- Supplementary Data - pdf file
