# Supplementary material for: Engineering a minimal G protein to facilitate crystallisation of G protein-coupled receptors in their active conformation
Source: Protein Eng Des Sel. 2016 Nov 28;29(12):583–94. doi: 10.1093/protein/gzw049 (PMC5181381; doi:10.1093/protein/gzw049)
Supplement: Supplementary Data [file supp_gzw049_PEDS_SIBC.pdf]

Supplementary material

Engineering a minimal G Protein to facilitate crystallisation of G protein-coupled receptors in their active conformation

Byron Carpenter & Christopher G. Tate

MRC Laboratory of Molecular Biology, Cambridge Biomedical Campus, Francis Crick Avenue, Cambridge, CB2 0QH, UK

Supplementary methods

Purification of non-lipidated Gα_s

The cell pellet from 6 L of insect cell culture was resuspended to 400 ml in buffer A (30 mM Tris pH 8.0, 100 mM NaCl, 5 mM $MgCl_2$, 5 mM imidazole, 50 μM GDP). PMSF (1 mM), Pepstatin-A (2.5 μM), Leupeptin (10 μM), Complete protease tablets (Roche), DNase I (50 μg/ml), and DTT (100 μM) were added to give the final concentrations indicated. Cells were broken by sonication (10 minutes at 70% amplitude) and clarified by centrifugation (38,000*g* for 1 h). The supernatant was loaded onto a 5 ml Ni Sepharose FF column (GE Healthcare) at 5 ml/min. The column was washed sequentially with 25 ml buffer A, 50 ml buffer B (20 mM Tris pH 8.0, 300 mM NaCl, 10 mM imidazole, 10% glycerol, 1 mM $MgCl_2$, 50 μM GDP), and 25 ml buffer C (20 mM Tris pH 8.0, 300 mM NaCl, 30 mM imidazole, 10% glycerol, 1 mM $MgCl_2$, 50 μM GDP) at 5 ml/min. The column was eluted with 25 ml buffer D (20 mM Tris pH 9.0, 50 mM NaCl, 500 mM imidazole, 10% glycerol, 1 mM $MgCl_2$, 50 μM GDP). The eluate was diluted to 250 ml in buffer E (20 mM Tris pH 9.0, 50 mM NaCl, 10% glycerol, 1 mM $MgCl_2$, 50 μM GDP, 1 mM DTT) and loaded onto a 5 ml Q Sepharose HP column (GE Healthcare) at 5 ml/min. The column was washed with 50 ml buffer E and eluted with a linear gradient of 50-300 mM NaCl (in buffer E). Peak fractions were pooled and TEV protease was added to give a final ratio of 1:20 w/w (TEV: Gα_s). The sample was dialysed overnight against 1 L buffer F (20 mM HEPES pH 7.5, 100 mM NaCl, 10% glycerol, 1 mM $MgCl_2$, 10 μM GDP). Imidazole (20 mM) and Ni-NTA resin (4 ml) were added to the sample and mixed for 1 h. The mixture was poured onto a disposable column containing 1 ml Ni-NTA resin, and the flow-through collected. The column was washed with 10 ml buffer F and this wash was pooled with the flow-through. The pooled sample was concentrated to 1.5 ml using a 10 kDa MWCO Amicon Ultra centrifugal filter (Millipore). The sample was loaded onto a Superdex-200 26/600 gel filtration column (GE Healthcare), equilibrated with buffer G (10 mM HEPES pH 7.5, 100 mM NaCl, 10% glycerol, 1 mM $MgCl_2$, 1 μM GDP, 0.1 mM TCEP). Peak fractions were pooled and concentrated to 50 mg/ml. The pure protein was aliquoted, flash-frozen in liquid nitrogen, and stored at -80°C. A typical yield was 6.5 mg of pure Gα_s per litre of culture.

Purification of non-lipidated G_s heterotrimer

Purification of non-lipidated heterotrimeric G_s ($G\alpha_s\beta_1\gamma_2$) was performed essentially as described for non-lipidated $G\alpha_s$, except: the Ni Sepharose column was washed with 50 ml buffer C, instead of 25 ml; buffer D contained 300 mM imidazole, instead of 500 mM; the pH of buffers D and E was 8.5, instead of 9.0; the Q Sepharose column was eluted with a linear gradient of 50-200 mM NaCl, instead of 50-300mM; and no TEV cleavage step was performed, instead, fractions from the Q Sepharose column were concentrated and loaded onto the Superdex-200 column. A typical yield was 7 mg of pure G_s per litre of culture.

Purification of non-lipidated Gβγ dimer

Purification of non-lipidated Gβγ dimer ($G\beta_1\gamma_2$) was performed essentially as described for non-lipidated $G\alpha_s$, except: GDP was omitted from all buffers; $MgCl_2$ was omitted from buffers B-G; buffers B and C contained 250 mM NaCl, instead of 300 mM; buffer D contained 25 mM NaCl, instead of 50 mM; buffer D contained 300 mM imidazole, instead of 500 mM; buffers E and F were supplemented with 1 mM EDTA; the Q Sepharose column was eluted with a linear gradient of 25-200 mM NaCl, instead of 50-300mM; and no TEV cleavage step was performed, instead, fractions from the Q Sepharose column were concentrated and loaded onto the Superdex-200 column. A typical yield was 7.5 mg of pure Gβγ per litre of culture.

Expression and purification of nanobodies

Nanobodies were expressed and purified using modified versions of previously described methods (Rasmussen *et al.*, 2011; Rasmussen *et al.*, 2011). *E. coli* strain BL21(DE3)RIL (Agilent Technologies) transformed with Nb80 or Nb35 DNA was grown in TB media supplemented with glucose (0.2%) and $MgSO_4$ (5 mM). Cultures were grown at 30°C until an OD_{600} of 0.8 was reached. Expression was induced with IPTG (200 μM for Nb80 or 50 μM for Nb35) and the temperature reduced to 25°C. Cells were harvested 20 h post-induction by centrifugation at 5000*g* for 10 mins, flash-frozen in liquid nitrogen and stored at -80°C.

The cell pellet from 6 L of Nb80 culture was resuspended to 300 ml in buffer H (20 mM HEPES pH 7.5, 100 mM NaCl, 10 mM imidazole, 5 mM $MgCl_2$). PMSF (1 mM), Complete protease tablets,

DNase I (50 μg/ml), and lysozyme (100 μg/ml) were added. Cells were broken by sonication (10 minutes at 70% amplitude) and clarified by centrifugation (38,000*g* for 30 mins). The supernatant was loaded onto a 5 ml Ni Sepharose FF column at 5 ml/min. The column was washed with 100 ml buffer I (20 mM HEPES pH 7.5, 500 mM NaCl, 40 mM imidazole) at 5 ml/min. The column was eluted with 20 ml buffer J (20 mM HEPES pH 7.5, 100 mM NaCl, 500 mM imidazole). The eluate was concentrated to 1.5 ml and loaded onto a Superdex-200 26/600 gel filtration column, equilibrated with buffer K (10 mM HEPES pH 7.5, 100 mM NaCl, 10% glycerol). Peak fractions were pooled and concentrated to 50 mg/ml. The pure protein was aliquoted, flash-frozen in liquid nitrogen, and stored at -80°C. A typical yield of Nb80 was 12 mg of pure protein per litre of culture.

The cell pellet from 6 L of Nb35 culture was resuspended to 300 ml in buffer L (20 mM HEPES pH 7.5, 100 mM NaCl, 5 mM imidazole, 5 mM $MgCl_2$). PMSF (1 mM), Complete protease tablets, DNase I (50 μg/ml), and lysozyme (100 μg/ml) were added to give the final concentrations indicated. Cells were broken by sonication (10 minutes at 70% amplitude) and clarified by centrifugation (38,000*g* for 30 mins). The supernatant was loaded onto a 5 ml Ni Sepharose FF column at 5 ml/min. The column was washed with 100 ml buffer I at 5 ml/min. The column was eluted with 20 ml buffer M (20 mM HEPES pH 7.0, 500 mM imidazole). The eluate was diluted to 200 ml in buffer N (20 mM HEPES pH 7.0) and loaded onto a 5 ml SP Sepharose HP column (GE Healthcare) at 5 ml/min. The column was washed with 50 ml buffer N and eluted with a linear gradient of 0-200 mM NaCl (in buffer N). Peak fractions were pooled, concentrated to 1.5 ml and loaded onto a Superdex-200 26/600 gel filtration column, equilibrated with buffer K. Peak fractions were pooled and concentrated to 50 mg/ml. The pure protein was aliquoted, flash-frozen in liquid nitrogen, and stored at -80°C. A typical yield of Nb35 was 26 mg of pure protein per litre of culture.

Expression and purification of mini-G_s mutants (for screening)

E. coli strain BL21(DE3)RIL transformed with mini-G_s DNA was grown in 2TY media supplemented with glucose (0.1%). Cultures were grown at 30°C until an OD_{600} of 0.8 was reached. Expression was induced with IPTG (100 μM) and the temperature reduced to 15°C. Cells were harvested 20 h post-induction by centrifugation at 5000*g* for 10 mins, flash-frozen in liquid nitrogen and stored at -80°C.

The cell pellet from 1 L of culture was resuspended in 20 ml in buffer O (40 mM HEPES pH 7.5, 100 mM NaCl, 20% glycerol, 10 mM imidazole, 5 mM $MgCl_2$, 3 mM ATP, 100 μM GDP). PMSF (1 mM),

Pepstatin-A (2.5 μM), Leupeptin (10 μM), Complete protease tablets, DNase I (20 μg/ml), lysozyme (50 μg/ml) and DTT (100 μM) were added to give the final concentrations indicated. Cells were broken by sonication (2 minutes at 70% amplitude) and clarified by centrifugation (50,000*g* for 20 mins). The supernatant was removed, 1 ml Ni Sepharose FF resin was added, and the sample was mixed for 90 mins at 4°C. The mixture was poured onto a disposable column and the resin was washed with 20 ml buffer P (20 mM HEPES pH 7.5, 500 mM NaCl, 20% glycerol, 40 mM imidazole, 1 mM $MgCl_2$, 50 μM GDP). The column was eluted with 2.5 ml buffer Q (20 mM HEPES pH 7.5, 100 mM NaCl, 20% glycerol, 400 mM imidazole, 1 mM $MgCl_2$, 50 μM GDP). The sample was exchanged into buffer R (20 mM HEPES pH 7.5, 100 mM NaCl, 10% glycerol, 1 mM $MgCl_2$, 10 μM GDP, 0.1 mM TCEP) using a PD10 column (GE Healthcare). The sample was concentrated to 100 μl, flash-frozen in liquid nitrogen, and stored at -80°C. Up to 20 mutants could be purified in parallel in a single day using this protocol.

Expression and purification of mini-G_s (final protocol)

E. coli strain BL21(DE3)RIL transformed with mini-G_s DNA was grown in TB media supplemented with glucose (0.2%), $MgSO_4$ (5 mM) and antifoam (0.01%). Cells were cultured in 2 L baffled flasks (Simax), shaking at 140 rpm. Cultures were grown at 30°C until an OD_{600} of 0.8 was reached. Expression was induced with IPTG (50 μM) and the temperature reduced to 25°C. Cells were harvested 20 h post-induction by centrifugation at 5000*g* for 10 mins, flash-frozen in liquid nitrogen and stored at -80°C.

The cell pellet from 1 L of culture was resuspended to 200 ml in buffer S (40 mM HEPES pH 7.5, 100 mM NaCl, 10% glycerol, 10 mM imidazole, 5 mM $MgCl_2$, 50 μM GDP). PMSF (1 mM), Pepstatin-A (2.5 μM), Leupeptin (10 μM), Complete protease tablets, DNase I (50 μg/ml), lysozyme (100 μg/ml) and DTT (100 μM) were added to give the final concentrations indicated. Cells were broken by sonication (10 minutes at 70% amplitude) and clarified by centrifugation (38,000*g* for 45 mins). The supernatant was loaded onto a 10 ml Ni Sepharose FF column at 5 ml/min. The column was washed with 100 ml buffer T (20 mM HEPES pH 7.5, 500 mM NaCl, 40 mM imidazole, 10% glycerol, 1 mM $MgCl_2$, 50 μM GDP) at 5 ml/min. The column was eluted with 30 ml buffer U (20 mM HEPES pH 7.5, 100 mM NaCl, 500 mM imidazole, 10% glycerol, 1 mM $MgCl_2$, 50 μM GDP). TEV protease was added to give a final ratio of 1:20 w/w (TEV: mini-G_s). DTT (1mM) was added and the sample was dialysed overnight against 2 L buffer V (20 mM HEPES pH 7.5, 100 mM NaCl, 10% glycerol, 1 mM $MgCl_2$, 10 μM GDP). Imidazole

(20 mM) and Ni-NTA resin (4 ml) were added to the sample and mixed for 1 h. The mixture was poured onto a disposable column containing 1m Ni-NTA resin, and the flow-through collected. The column was washed with 10 ml buffer V and this wash was pooled with the flow-through. The pooled sample was concentrated to 1.5 ml and loaded onto a Superdex-200 26/600 gel filtration column, equilibrated with buffer W (10 mM HEPES pH 7.5, 100 mM NaCl, 10% glycerol, 1 mM $MgCl_2$, 1 μM GDP, 0.1 mM TCEP). Peak fractions were pooled and concentrated to 100 mg/ml. The pure protein was aliquoted, flash-frozen in liquid nitrogen, and stored at -80°C. A typical yield was 100 mg pure protein per litre of culture.

Supplementary Table SI. Non-lipidated G protein constructs used during this work

Construct	Subunit	Isoform	Species	Deleted residues	Mutation	Other modifications
Soluble $G\alpha_s$	$G\alpha_s$	Long	Human	1-5		10 His tag (N-term)<br>TEV protease site
Soluble $G\beta\gamma$	$G\beta_1$		Human			8 His Tag (N-term)
	$G\gamma_2$		Human		C68S	
Soluble G_s	$G\alpha_s$	Long	Human	1-5		
	$G\beta_1$		Human			8 His tag (N-term)
	$G\gamma_2$		Human		C68S	

Long isoform human $G\alpha_s$ incorporated a five amino acid deletion at the N-terminus to prevent post-translational lipidation. $G\gamma_2$ incorporated the C68S mutation to prevent post-translational lipidation. The $G\beta\gamma$ dimer and G_s heterotrimer incorporated a histidine tag on only the β subunit, to facilitate purification of intact complexes.

Supplementary Table SII. Turkey β_1AR constructs used during this work

Construct	Deleted residues	Mutations	Other modifications
$\beta_1AR^{\Delta NC}$	1-32<br>424-483	C116L	6 His tag (C-term)
β_1AR-84	1-32<br>244-271<br>368-483	M40C<br>M90V<br>L103C<br>C116L<br>D322K<br>F327A<br>C358A<br>F388M	MBP fusion (N-term)<br>6 His tag (C-term)

The $\beta_1AR^{\Delta NC}$ construct contained N-terminal and C-terminal truncations; and the C116L mutation. These modifications were designed to prevent N-glycosylation and/or improve expression (Warne *et al.*, 2003). The β_1AR-84 construct contained: a deletion of cytoplasmic loop 3; thermostabilising mutations M90V, D322K, F327A, and F388M (Miller-Gallacher *et al.*, 2014; Serrano-Vega *et al.*, 2008); a disulphide link between transmembrane helices 1 and 2, facilitated by the M40C and L103C mutations (Miller-Gallacher*, et al.*, 2014); the C358A mutation, designed to prevent palmitoylation; and an N-terminal MBP fusion.

Supplementary Table SIII. Mini-G_s constructs used during this work

Construct	Deleted residues	GαAH linker	Mutations	Other modifications
Mini-G_s77	1-21<br>67-193	Gly_3	L197A<br>C200S	N-terminal 6 His tag
Mini-G_s161	1-21<br>65-208	Gly_5	None	N-terminal 6 His tag
Mini-G_s199	1-21<br>65-203<br>255-264 (switch III)	Gly_8	G49D<br>E50N<br>A249D<br>S252D<br>L272D	N-terminal 6 His tag
Mini-G_s391	1-25<br>65-203<br>255-264 (switch III)	GGSGGSGG	G49D<br>E50N<br>A249D<br>S252D<br>L272D<br>I372A	N-terminal 6 His tag<br>TEV protease site
Mini-G_s393	1-25<br>65-203<br>255-264 (switch III)	GGSGGSGG	G49D<br>E50N<br>A249D<br>S252D<br>L272D<br>I372A<br>V375I	N-terminal 6 His tag<br>TEV protease site
Mini-G_s399	1-5<br>65-203<br>255-264 (switch III)	GGSGGSGG	G49D<br>E50N<br>A249D<br>S252D<br>I372A<br>V375I	N-terminal 6 His tag<br>TEV protease site
Mini-G_s404	1-25<br>65-203<br>255-264 (switch III)	GGSGGSGG	G49D<br>E50N<br>A249D<br>S252D<br>L272D	N-terminal 6 His tag<br>TEV protease site

Mini-G_s77 contained mutations of two residues (L197A and C200S), which were buried in $G\alpha_s$, but became surface exposed after deletion of the GαAH domain. These mutations helped to improve expression and reduce aggregation of mini-G_s77. The mutations were located in a region of switch I, which was later deleted, so they do not appear in subsequent constructs

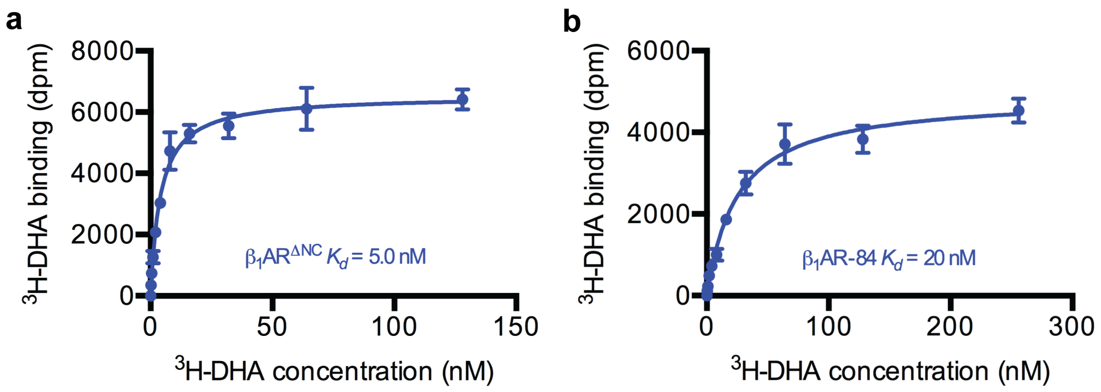

Supplementary Fig. S1 Saturation binding data for β_1AR constructs. (**a**) The apparent dissociation constant (K_d) of ^{3}H-dihydroalprenolol (^{3}H-DHA) for β_1AR$^{\Delta NC}$ was 5.0 ± 0.6 nM. (**b**) The apparent K_d of ^{3}H-DHA for β_1AR-84 was 20 ± 3 nM. K_d values represent mean ± SEM of three independent experiments, each performed in duplicate. Curves shown are from a representative experiment.

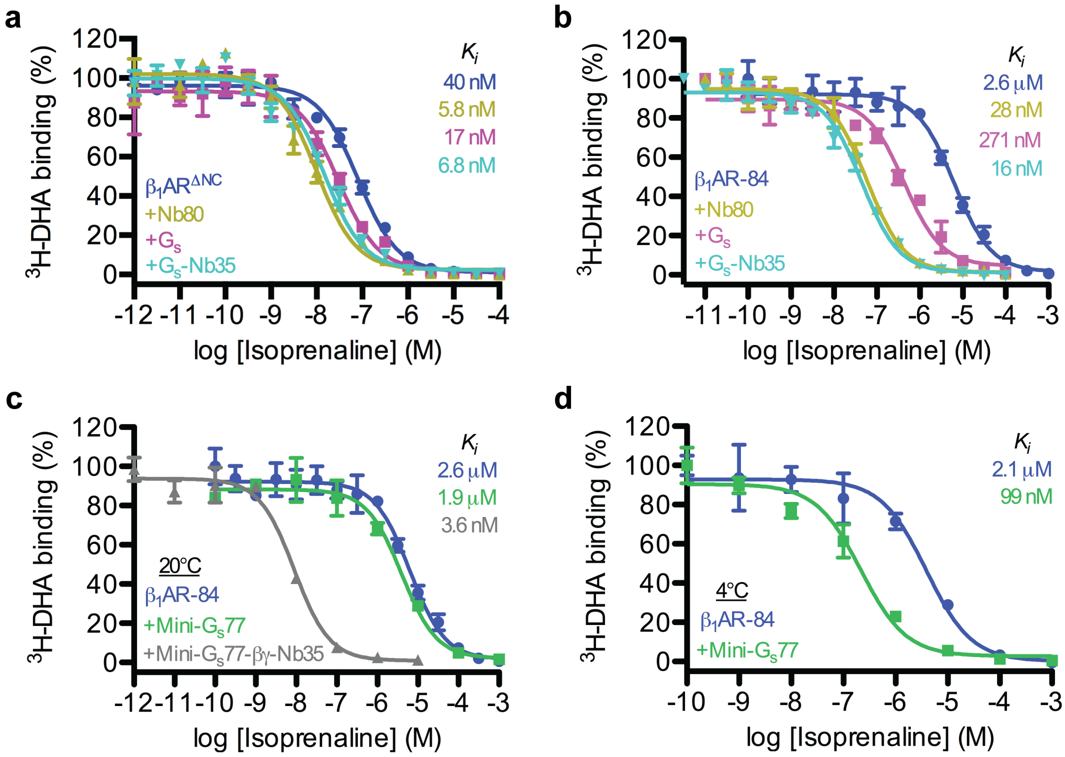

Supplementary Fig. S2 Measuring G protein coupling to membrane-embedded β_1AR using a competition binding assay. (**a**) Binding of Nb80, G_s, or G_s–Nb35 to β_1AR$^{\Delta NC}$ induced a shift in isoprenaline (agonist) affinity (K_i) from 40 ± 0 nM (n=2) to 5.8 ± 0.8 nM (n=2), 17 ± 2 nM (n=2), or 6.8 ± 0.6 nM (n=2), respectively. (**b**) Binding of Nb80, G_s, or G_s–Nb35 to β_1AR-84 induced a shift in isoprenaline K_i from 2.6 ± 0.3 μM (n=15) to 28 ± 1 nM (n=2), 271 ± 54 nM (n=2), or 16 ± 4 nM (n=3), respectively. (**c**) Binding of Mini-G_s77 to β_1AR-84 at 20°C did not induce a significant shift in isoprenaline K_i (2.6 ± 0.3 μM (n=15) *versus* 1.9 ± 0.2 μM (n=3); P = 0.254). Binding of Mini-G_s77–βγ–Nb35 to β_1AR-84 at 20°C induced a shift in isoprenaline K_i from 2.6 ± 0.3 μM (n=15) to 3.6 ± 0.8 nM (n=2). (**d**) Binding of Mini-G_s77 to β_1AR-84 at 4°C induced a shift in isoprenaline K_i from 2.1 ± 0.2 μM (n=12) to 99 ± 12 nM (n=4). K_i values represent mean ± SEM from the number (n) of independent experiments indicated, each performed in duplicate. Curves shown are from a representative experiment.

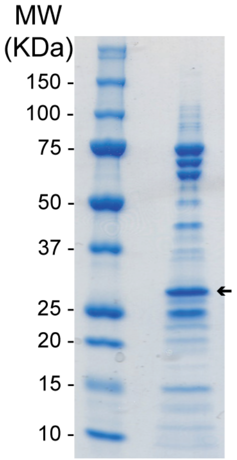

Supplementary Fig. S3 SDS-PAGE analysis of mini-G_s77 purification. Mini-G_s77 (indicate by the arrow) could be partially purified with a yield of approximately 200 μg per litre of *E. coli* culture, and purity of approximately 10-20%.

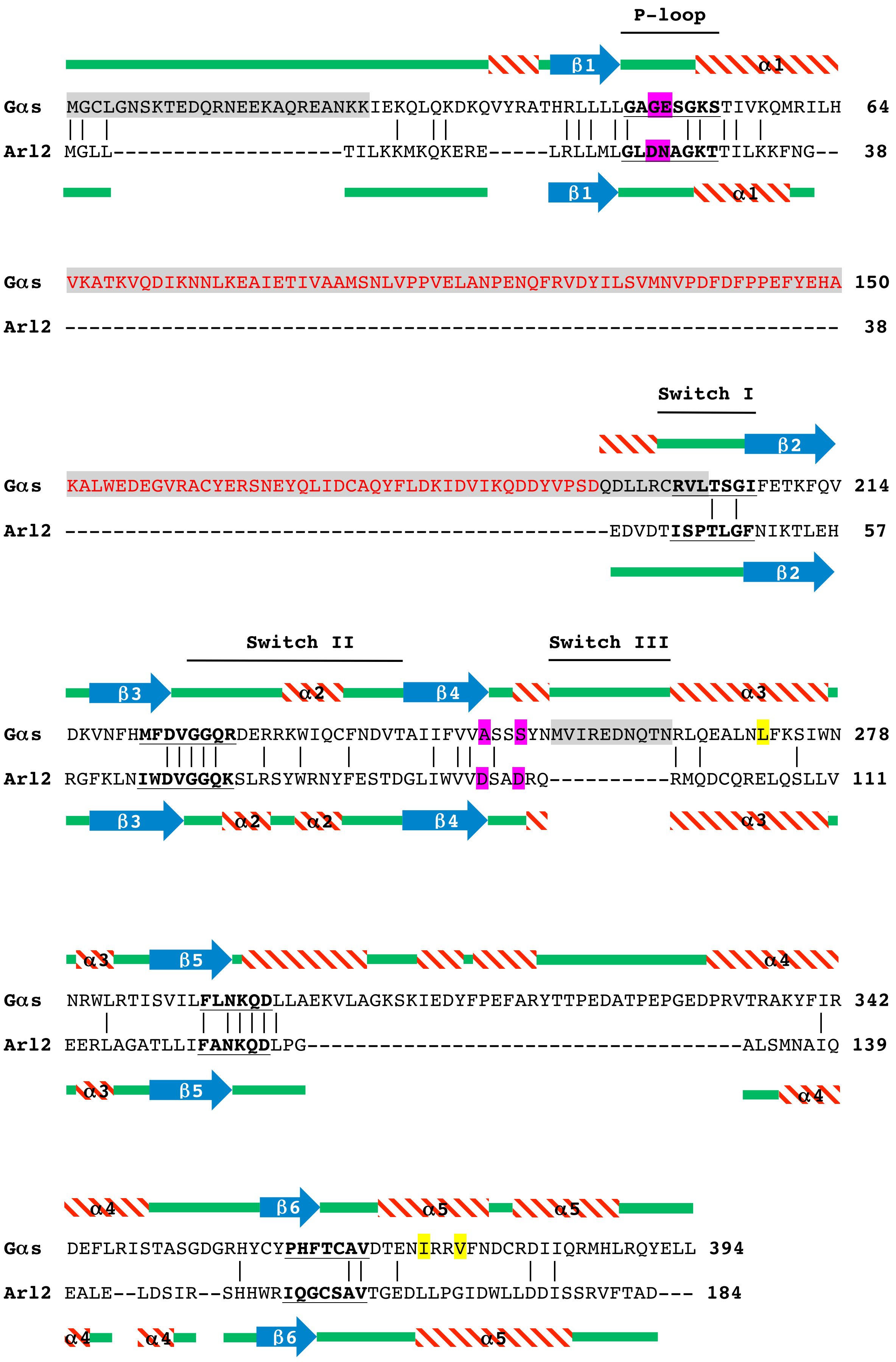
P-loop
β1
α1
Gαs MGCLGNSKTEDQRNEEKAQREANKKIEKQLQKDKQVYRATHRLLLLGAGESGKSTIVKQMRILH 64
Arl2 MGLL--------------------TILKKMKQKERE-----LRLLMLGLDNAGKTTILKKFNG-- 38
β1
α1
Gαs VKATKVQDIKNNLKEAIETIVAAMSNLVPPVELANPENQFRVDYILSVMNVPDFDFPPEFYEHA 150
Arl2 -- 38
Switch I
β2
Gαs KALWEDEGVRACYERSNEYQLIDCAQYFLDKIDVIKQDDYVPSDQDLLRCRVLTSGIFETKFQV 214
Arl2 --EDVDTISPTLGFNIKTLEH 57
β2
Switch II
Switch III
β3
α2
β4
α3
Gαs DKVNFHMFDVGGQRDERRKWIQCFNDVTAIIFVVASSSYNMVIREDNQTNRLQEALNLFKSIWN 278
Arl2 RGFKLNIWDVGGQKSLRSYWRNYFESTDGLIWVVDSADRQ----------RMQDCQRELQSLLV 111
β3
α2
α2
β4
α3
α3
β5
α4
Gαs NRWLRTISVILFLNKQDLLAEKVLAGKSKIEDYFPEFARYTTPEDATPEPGEDPRVTRAKYFIR 342
Arl2 EERLAGATLLIFANKQDLPG------------------------------------ALSMNAIQ 139
α3
β5
α4
α4
β6
α5
α5
Gαs DEFLRISTASGDGRHYCYPHFTCAVDTENIRRVFNDCRDIIQRMHLRQYELL 394
Arl2 EALE--LDSIR--SHHWRIQGCSAVTGEDLLPGIDWLLDDISSRVFTAD--- 184
α4
α4
β6
α5

Supplementary Fig. S4 Sequence alignment between Gα_s (PDB code 1AZT; NCBI accession NP_851364) (Sunahara *et al.*, 1997) and Arl2 (PDB code 1KSH; NCBI accession NP_062696) (Hanzal-Bayer *et al.*, 2002), performed using the program DALI (Holm and Rosenstrom, 2010). Secondary structure features are shown as: loop, green bar; α-helix, red striped box; and β-strand, blue arrow. Secondary structure elements common to both proteins are labelled using the naming convention for G proteins (Sprang, 1997). Mini-G$_s$ mutations that were designed based on the structure of Arl2 (Hanzal-Bayer*, et al.*, 2002) are highlighted in magenta, other mutations are highlighted in yellow, and deletions are highlighted in grey. The helical domain is shown in red and secondary structure elements have been omitted for clarity. The switch regions and P-loop are indicated and the five G-box motifs (Sprang, 1997) are shown in bold and underlined.

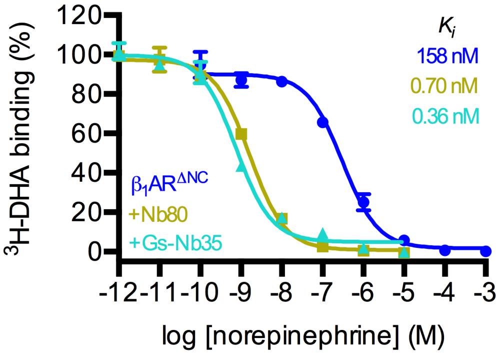

Supplementary Fig. S5 $\beta_1AR^{\Delta NC}$ competition binding assay using the agonist norepinephrine. The assay was performed under identical buffer conditions used for the thermostability assay. Binding of Nb80 or G_s–Nb35 to $\beta_1AR^{\Delta NC}$ induced a shift in norepinephrine K_i from 158 ± 6 nM to 0.70 ± 0.11 nM or 0.36 ± 0.02 nM, respectively. K_i values represent mean ± SEM of two independent experiments, each performed in duplicate. Curves shown are from a representative experiment.

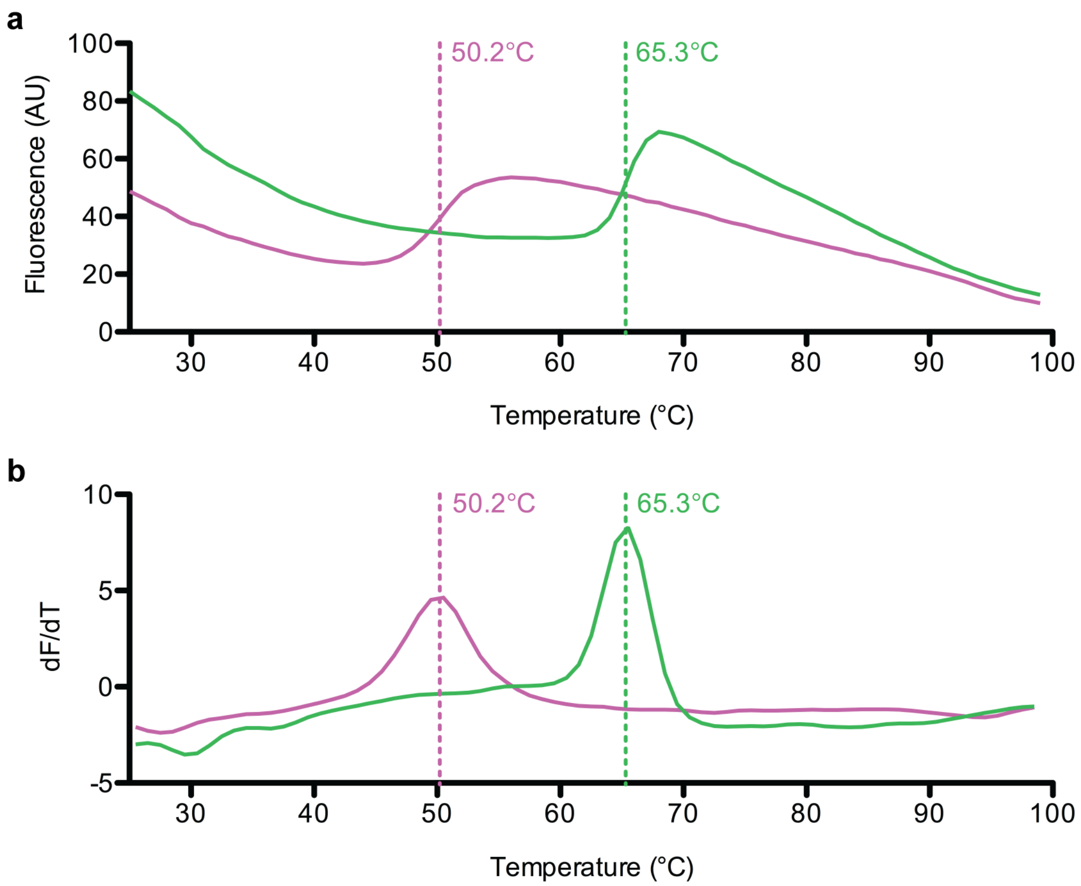

Supplementary Fig. S6 Examples of T_m data obtained from differential scanning fluorimetry experiments, for Gα_s (magenta) and mini-G_s393 (green). (**a**) Apparent T_m values correspond to the inflection point of the curve. (**b**) Clearer visualisation of melting curves was achieved by plotting the derivative of fluorescence over temperature (dF/dT) *versus* temperature. Curves shown are from a representative experiment, performed in duplicate.

```
Gαs       MGCLGNSKTEDQRNEEKAQREANKKIEKQLQKDKQVYRATHRLLLLGAGESGKSTIVKQM   60
Mini-Gs   ------------------------GIEKQLQKDKQVYRATHRLLLLGADNSGKSTIVKQM   60
                                   ***********************  **********

Gαs       RILHVNGFNGEGGEEDPQAARSNSDGEKATKVQDIKNNLKEAIETIVAAMSNLVPPVELA  120
Mini-Gs   RILHGGSGGSGG------------------------------------------------  120
          ****

Gαs       NPENQFRVDYILSVMNVPDFDFPPEFYEHAKALWEDEGVRACYERSNEYQLIDCAQYFLD  180
Mini-Gs   ------------------------------------------------------------  180

Gαs       KIDVIKQADYVPSDQDLLRCRVLTSGIFETKFQVDKVNFHMFDVGGQRDERRKWIQCFND  240
Mini-Gs   -----------------------TSGIFETKFQVDKVNFHMFDVGGQRDERRKWIQCFND  240
                                 *************************************

Gαs       VTAIIFVVASSSYNMVIREDNQTNRLQEALNLFKSIWNNRWLRTISVILFLNKQDLLAEK  300
Mini-Gs   VTAIIFVVDSSDY----------NRLQEALNDFKSIWNNRWLRTISVILFLNKQDLLAEK  300
          ******** ** *          ******** ****************************

Gαs       VLAGKSKIEDYFPEFARYTTPEDATPEPGEDPRVTRAKYFIRDEFLRISTASGDGRHYCY  360
Mini-Gs   VLAGKSKIEDYFPEFARYTTPEDATPEPGEDPRVTRAKYFIRDEFLRISTASGDGRHYCY  360
          ************************************************************

Gαs       PHFTCAVDTENIRRVFNDCRDIIQRMHLRQYELL  394
Mini-Gs   PHFTCAVDTENARRIFNDCRDIIQRMHLRQYELL  394
          *********** ** *******************
```

Supplementary Fig. S7 Sequence alignment between $G\alpha_s$ (NCBI accession NP_851364) and mini-G_s393, showing the mutations and deletions that were incorporated into mini-G_s. Mutations are highlighted in grey, and deletions are indicated by dashes, the linker used to connect the termini of the GTPase domain (after deletion of the helical domain) is highlighted in turquoise. The sequence of mini-G_s393 presented is that of the final purified protein after removal of the histidine tag and so it contains one exogenous glycine residue at the N-terminus (bold) from the TEV cleavage site. The sequence of the mini-G_s393 expression construct is given in Supplementary Fig. S8.

Mini-G_s393 gene sequence

CC**ATG**GGTCACCACCATCATCACCATGAAAATCTTTATTTCCAGGGTATCGAGAAGCAGCTGCAGAAGGACAAGCA
GGTCTACCGGGCCACGCACCGCCTGCTGCTGCTGGGTGCTG**ATAAT**TCTGGTAAAAGCACCATTGTGAAGCAGATG
AGGATCCTGCATGGTGGGAGTGGCGGGAGCGGAGGTACTTCTGGAATCTTTGAGACCAAGTTCCAGGTGGACAAAG
TCAACTTCCACATGTTTGACGTGGGTGGCCAGCGCGATGAACGCCGCAAGTGGATCCAGTGCTTCAACGATGTGAC
TGCCATCATCTTCGTGGTGG**A**CAGCAGC**GAT**TACAACCGCCTGCAGGAGGCTCTGAAC**GA**CTTCAAGAGCATCTGG
AACAACAGATGGCTGCGCACCATCTCTGTGATCCTGTTCCTCAACAAGCAAGATCTGCTCGCTGAGAAAGTCCTTG
CTGGGAAATCGAAGATTGAGGACTACTTTCCAGAATTTGCTCGCTACACTACTCCTGAGGATGCTACTCCCGAGCC
CGGAGAGGACCCACGCGTGACCCGGGCCAAGTACTTCATTCGAGATGAGTTTCTGAGGATCAGCACTGCCAGTGGA
GATGGGCGTCACTACTGCTACCCTCATTTCACCTGCGCTGTGGACACTGAGAAC**GC**CCGCCGT**ATC**TTCAACGACT
GCCGTGACATCATTCAGCGCATGCACCTTCGTCAGTACGAGCTGCTCTAATAGCTCGAG

Mini-G_s393 protein sequence

MGHHHHHHENLYFQGIEKQLQKDKQVYRATHRLLLLGA**DN**SGKSTIVKQMRILHGGSGGSGGTSGIFETKFQVDKV
NFHMFDVGGQRDERRKWIQCFNDVTAIIFVV**D**SS**D**YNRLQEALN**D**FKSIWNNRWLRTISVILFLNKQDLLAEKVLA
GKSKIEDYFPEFARYTTPEDATPEPGEDPRVTRAKYFIRDEFLRISTASGDGRHYCYPHFTCAVDTEN**A**RR**I**FNDC
RDIIQRMHLRQYELL

Supplementary Fig. S8 Sequence of mini-G_s393. The histidine tag is highlighted in red, the TEV protease cleavage site is highlighted in grey, and the linker used to replace the $G\alpha$AH domain is highlighted in turquoise. Mutations are shown in bold type and underlined. This construct was cloned into the pET15b vector using NcoI (yellow) and XhoI (magenta) restriction sites for *E. coli* expression. Stop codons are highlighted in green.

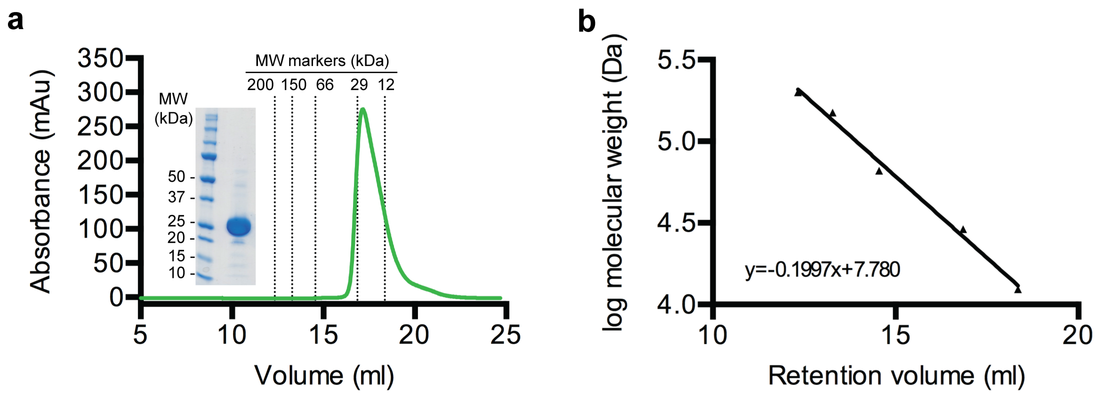

Supplementary Fig. S9 Gel filtration and SDS-PAGE analysis of mini-G_s393 purification. (**a**) Mini-G_s393 was purified with a yield of 100 mg per litre of *E. coli* culture, and could be concentrated to over 100 mg/ml. Pure mini-G_s393 resolved as a single peak with an apparent molecular weight of 22 kDa (retention volume of 17.2 ml) by analytical gel filtration chromatography. (**b**) Calibration curve used to calculate protein molecular weights from gel filtration profiles (see materials and methods). The protein standards used to calibrate the column were: cytochrome c (12.4 kDa), carbonic anhydrase (29 kDa), albumin (66 kDa), alcohol dehydrogenase (150 kDa) and β-amylase (200 kDa).

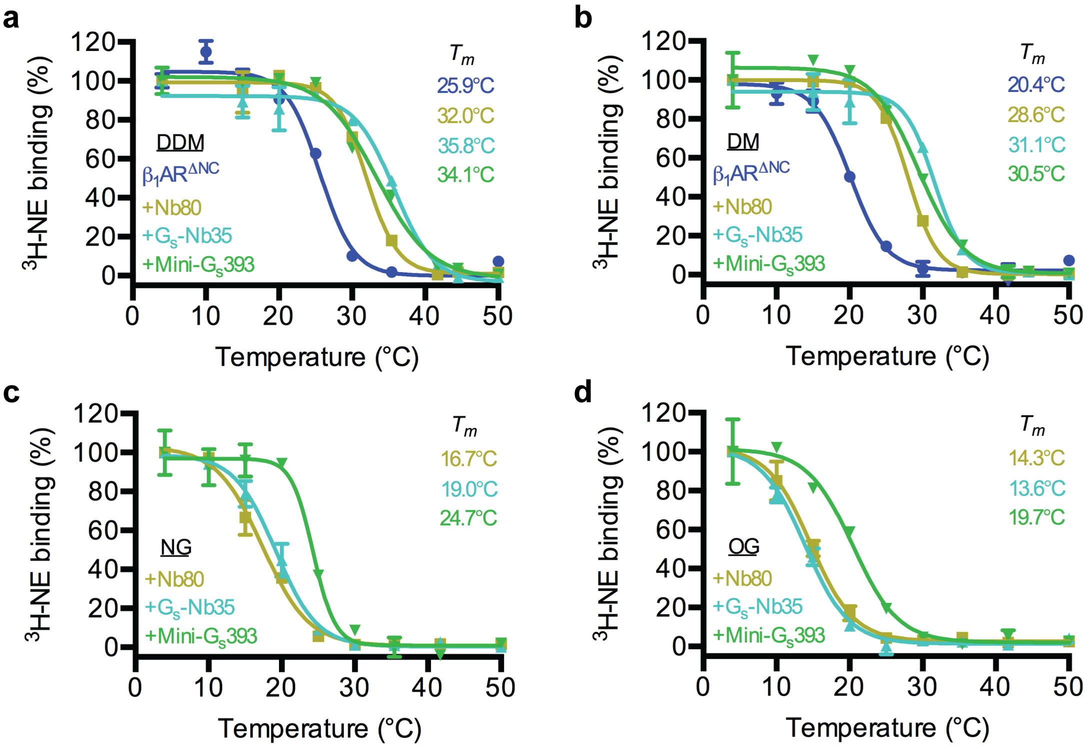

e

Binding partner	β_1AR$^{\Delta NC}$ apparent T_m (°C)			
	**DDM**	**DM**	**NG**	**OG**
None	25.9 ± 0.0 (n=3)	20.4 ± 0.4 (n=3)	No signal	No signal
Nb80	32.0 ± 0.0 (n=3)	28.6 ± 0.3 (n=3)	16.7 ± 0.7 (n=2)	14.3 ± 0.2 (n=3)
G_s–Nb35	35.8 ± 0.1 (n=3)	31.1 ± 0.4 (n=3)	19.0 ± 0.2 (n=2)	13.6 ± 0.2 (n=3)
Mini-G_s393	34.1 ± 0.5 (n=3)	30.5 ± 0.4 (n=3)	24.7 ± 0.4 (n=2)	19.7 ± 0.5 (n=3)

Supplementary Fig. S10 Thermostability data for β_1AR$^{\Delta NC}$ complexes. (**a-d**) Examples of T_m curves in different detergents: (**a**) DDM; (**b**) DM; (**c**) NG; (**d**) OG. (**e**) Table of compiled T_m data. Apparent T_m values represent mean ± SEM from the number (n) of independent experiments indicated, each performed in duplicate. Curves shown are from a representative experiment.

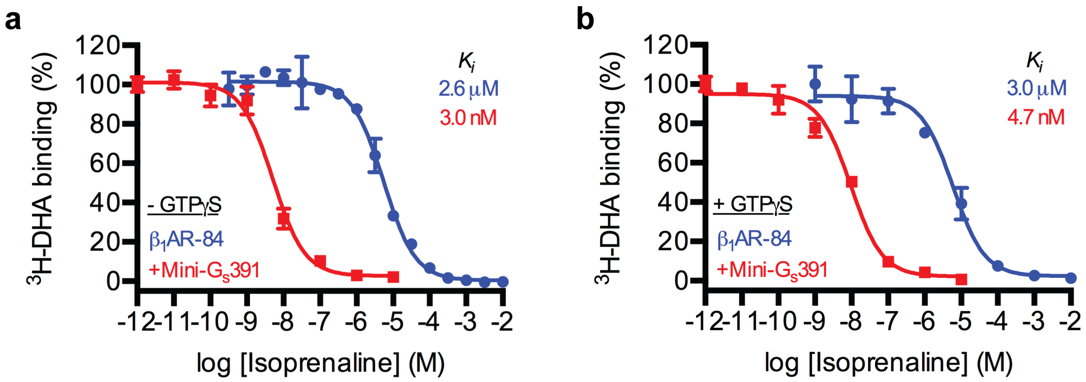

Supplementary Fig. S11 GTP-mediated dissociation of the β_1AR-84–mini-G_s391 complex, measured by competition binding assay. (**a**) In the absence of GTPγS, binding of mini-G_s391 (Supplementary Table SIII) to membrane-embedded β_1AR-84 induced a shift in isoprenaline K_i from 2.6 ± 0.3 μM (n=15) to 3.0 ± 0.4 nM (n=2). (**b**) In the presence of GTPγS (0.25 mM) binding of mini-G_s391 to membrane-embedded β_1AR-84 induced a shift in isoprenaline K_i from 3.0 ± 0.1 μM (n=2) to 4.7 ± 0.1 nM (n=2). There was no statistical difference in the isoprenaline K_i of the β_1AR-84–mini-G_s391 complex in the presence or absence of GTPγS (P = 0.053). K_i values represent mean ± SEM from the number (n) of independent experiments indicated, each performed in duplicate. Curves shown are from a representative experiment.

Supplementary references

Hanzal-Bayer, M., Renault, L., Roversi, P., Wittinghofer, A. and Hillig, R.C. (2002) *EMBO J,* **21**, 2095-2106.

Holm, L. and Rosenstrom, P. (2010) *Nucleic Acids Res,* **38**, W545-549.

Miller-Gallacher, J.L., Nehme, R., Warne, T., Edwards, P.C., Schertler, G.F., Leslie, A.G. and Tate, C.G. (2014) *PLoS One,* **9**, e92727.

Rasmussen, S.G., Choi, H.J., Fung, J.J., Pardon, E., Casarosa, P., Chae, P.S., Devree, B.T., Rosenbaum, D.M., Thian, F.S., Kobilka, T.S. *et al.* (2011) *Nature,* **469**, 175-180.

Rasmussen, S.G., DeVree, B.T., Zou, Y., Kruse, A.C., Chung, K.Y., Kobilka, T.S., Thian, F.S., Chae, P.S., Pardon, E., Calinski, D. *et al.* (2011) *Nature,* **477**, 549-555.

Serrano-Vega, M.J., Magnani, F., Shibata, Y. and Tate, C.G. (2008) *Proc Natl Acad Sci U S A,* **105**, 877-882.

Sprang, S.R. (1997) *Annu Rev Biochem,* **66**, 639-678.

Sunahara, R.K., Tesmer, J.J., Gilman, A.G. and Sprang, S.R. (1997) *Science,* **278**, 1943-1947.

Warne, T., Chirnside, J. and Schertler, G.F. (2003) *Biochim Biophys Acta,* **1610**, 133-140.
